# Measuring public opinion and acceptability of prevention policies: an integrative review and narrative synthesis of methods

**DOI:** 10.1186/s12961-022-00829-y

**Published:** 2022-03-04

**Authors:** Eloise Howse, Katherine Cullerton, Anne Grunseit, Erika Bohn-Goldbaum, Adrian Bauman, Becky Freeman

**Affiliations:** 1 The Australian Prevention Partnership Centre, Sax Institute, Sydney, Australia; 2 Prevention Research Collaboration, Sydney School of Public Health, Faculty of Medicine and Health, The University of Sydney, Sydney, Australia; 3 School of Public Health, Faculty of Medicine, University of Queensland, Brisbane, Australia

**Keywords:** Noncommunicable disease, Prevention, Review, Public opinion, Attitudes, Acceptability

## Abstract

Acceptability of and public support for prevention are an important part of facilitating policy implementation. This review aims to identify, summarize and synthesize the methods and study designs used to measure and understand public opinion, community attitudes and acceptability of strategies to prevent chronic noncommunicable disease (NCDs) in order to allow for examination of imbalances in methodological approaches and gaps in content areas. We searched four scientific databases (CINAHL, Embase, Ovid/MEDLINE and Scopus) for peer-reviewed, English-language studies published between January 2011 and March 2020 in high-income, democratic countries across North America, Europe and the Asia–Pacific region. Studies were included if they focused on opinions, attitudes and acceptability of primary prevention strategies and interventions addressing the key NCD risk factors of alcohol use, unhealthy diet, overweight/obesity, tobacco use and smoking, and physical inactivity. A total of 293 studies were included. Two thirds of studies (*n* = 194, 66%) used quantitative methods such as cross-sectional studies involving surveys of representative (*n* = 129, 44%) or convenience (*n* = 42, 14%) samples. A smaller number of studies used qualitative methods (*n* = 60, 20%) such as focus groups (*n* = 21, 7%) and interviews (*n* = 21, 7%). Thirty-nine studies (13%) used mixed methods such as content analysis of news media (*n* = 17, 6%). Tobacco control remains the dominant topic of public opinion literature about prevention (*n* = 124, 42%). Few studies looked solely at physical inactivity (*n* = 17, 6%). The results of this review suggest that public opinion and acceptability of prevention in the peer-reviewed literature is investigated primarily through cross-sectional surveys. Qualitative and mixed methods may provide more nuanced insights which can be used to facilitate policy implementation of more upstream strategies and policies to prevent NCDs.

**Supplementary Information:**

The online version contains supplementary material available at 10.1186/s12961-022-00829-y.

## Background

Noncommunicable diseases (NCDs) including cancers, diabetes and cardiovascular disease, are the leading causes of premature mortality and morbidity globally [1–3]. Key risk factors for NCDs include high systolic blood pressure, unhealthy dietary risks, tobacco use, high fasting plasma glucose, high body mass index, high cholesterol, alcohol use and physical inactivity [4]. In 2017 these risk factors contributed to 41.1 million deaths globally (73.5% of all deaths) [5].

Primary prevention of chronic disease focuses on addressing “upstream” environmental and social conditions that support good health across all populations and intervening before health risks and disease occur [6–8]. While some primary prevention interventions may target individuals, such as providing information for smoking cessation, other preventive interventions involve population-level strategies that target upstream policy, systems or environment (PSE) changes.

PSE change strategies create an enabling environment or system to facilitate healthier behaviours [9–11]. Preventive interventions addressing major disease risk factors, such as overweight and obesity, physical inactivity and poor diet, ultimately require multiple changes at different levels of the system to support improvements in health [12–14]. These strategies and interventions have also been described as “low-agency” interventions, referring to the reduced reliance on conscious action of individuals to engage with healthier behaviour [15], and “optimal defaults” or healthy “choice contexts”, where the healthier choice is the default option [16–19].

PSE change strategies for chronic disease prevention include measures such as mandatory nutrition labelling, sugary drinks taxes and restrictions on unhealthy food advertising [20, 21]; active transport infrastructure to promote cycling and walking [22]; minimum unit pricing of alcohol [23]; and plain packaging of tobacco products and tobacco taxes [24, 25], plus “endgame” strategies that focus on a tobacco-free future [26].

Despite the evidence for effectiveness and cost-effectiveness of many of these strategies [27], chronic disease prevention remains challenging to implement. While there has been some success globally in tobacco control through regulations like the WHO Framework Convention on Tobacco Control (FCTC) [28], governments have struggled to implement interventions in other areas [29]. This is particularly the case for interventions that involve the regulation of other harmful products and industries [30]. Previous research suggests there is less support for more population-wide, environmental and regulatory measures even though they are more effective in preventing NCDs [31–33].

One barrier to implementing and sustaining any intervention in public health is the acceptability of that intervention to the target population—a key process measure within public health evaluation models [34]. Measuring the acceptability of a proposed strategy or intervention can help to identify and understand the barriers and enablers for change while helping to mobilize support for greater preventive action [35]. Decision-makers and political representatives may also be more strongly influenced by the public’s opinion than by public health evidence [36, 37].

Previous reviews regarding opinion and attitudes about policies and interventions to prevent chronic disease have focused on populations and outcomes. A wide-ranging review by Diepeveen et al. [32] suggested some differences between population groups and attitudes regarding preventive interventions, including that women tend to be more supportive than men, while there was a mixed effect for age. This review also found that there is large body of research on attitudes towards tobacco control, with much of this work coming from the United States.

Other reviews of public opinion about prevention of NCDs are concentrated in tobacco control [38–40], alcohol policy [31, 41, 42] and obesity prevention [21, 43, 44]. Reviews have also been published that examine attitudes regarding health taxes [25], sugar-sweetened beverage (SSB) taxes [45], acceptability regarding financial incentives for health-related behaviour change, [46–48], opinions on sporting food environments [49], views of the Australian population on nutrition interventions [33] and the role of the media in framing public policies to prevent chronic disease [50, 51]. No reviews have been identified regarding attitudes towards physical activity interventions.

While these reviews indicate there is a substantial body of published evidence about acceptability of prevention, few, if any, reviews reflect on the methods and study designs used to collect and analyse public opinion and attitude data. Study design, question and hypothesis framing, methods (including sampling methods), response rates and types of analyses can affect perceived differences in views within and across populations, settings and countries. Furthermore, quantitative and qualitative methods are both used to understand public opinion about prevention, but the use of these methods has different purposes; these methods therefore demonstrate different types of evidence that can be used to inform policy-making and implementation of preventive interventions and policies.

### Review aims

This review aims to:

1. identify, summarize and synthesize the research methods and study designs used to measure public opinion, community attitudes and acceptability of PSE change strategies and policies to prevent NCDs; and
2. consider the implications of different methods, including new and emerging methods, and discuss the challenges, gaps and opportunities of using specific methods.

## Methods

### Review type

We considered that an integrative review using systematic search methods and narrative synthesis was appropriate given the heterogeneity of the phenomena under investigation, the broad range of PSE change strategies and risk factors included, and the inclusion of both quantitative and qualitative methodologies with diverse study designs [52]. Our approach could also be characterized as a critical review using a comprehensive and systematic search process that produces a synthesis of evidence on a topic [53]. PRISMA [Preferred Reporting Items for Systematic Reviews and Meta-Analyses] guidelines were followed and reported on to ensure that a systematic research process was used (see Additional file 1 for the reporting checklist).

### Search terms

Search terms were developed, tested and finalized by the first author in consultation with the other coauthors and an expert public health librarian. Full search terms and history can be found in Additional file 2.

### Database search

Four scientific databases were searched between October 2019 and March 2020: CINAHL, Embase, Ovid (MEDLINE) and Scopus. Search results can be found in Additional file 2.

### Inclusion and exclusion criteria

Table 1 summarizes the inclusion and exclusion criteria for the review.

**Table 1:** Inclusion and exclusion criteria

	Inclusion	Exclusion
Publication date range	Published between January 2011 and March 2020	Published prior to 2011 or after March 2020
Language	English	Non-English language
Countries	High-income, democratic countries in the Asia–Pacific, Europe (including Scandinavia) and the European Union, North America, Great Britain and/or OECD Cross-country studies such as the ITC Study (including the above countries or pan-Europe)	Nondemocratic countries Low- or middle-income countries Countries outside the regions specified
Type of publication	Original studies or empirical research published in peer-reviewed journals	Letters Editorials Commentary Opinion pieces Essays Reviews Protocols Grey literature Unpublished research
Type of research	Quantitative Qualitative Mixed methods	
Study design	Representative cross-sectional survey Nonrepresentative (convenience) cross-sectional survey Longitudinal or serial study Focus group Interviews Media analysis Deliberative study (e.g. citizen jury) Consultations / Delphi processes Other	Experimental studies (e.g., framing effects) Systematic or other type of review Meta-analyses
Population	General population (“public”/citizens) Children or adolescents Adults Staff/employees Students Academics Policy-makers and practitioners Politicians/representatives	Patients Healthcare professionals and clinicians e.g., doctors, pharmacists
Risk factors	Improve diet, food or nutrition, including sugar-sweetened beverages Physical inactivity Alcohol use Overweight and obesity Tobacco use or smoking	Mental health and suicide Illicit drugs Injury prevention not linked to the included risk factors Other public health topics (e.g. abortion, vaccination, sexual health/HIV) Breastfeeding E-cigarettes and tobacco cessation practices
Main outcomes measured by study	Opinion, view, attitude, belief or support regarding primary prevention of lifestyle-related chronic disease, such as laws, regulation, policies, taxation, labelling, restrictions, bans, government intervention, or any other PSE change strategy	Attitudes or beliefs about health, risk factors, diseases, conditions or treatment Opinions or attitudes about secondary or tertiary prevention of chronic disease (such as views on weight loss interventions, smoking cessation/e-cigarettes, and pharmaceutical interventions) Attitudes or beliefs relating to a process evaluation (implementation) of a programme or intervention Health promotion or health education practices and programmes

*ITC* International Tobacco Control Study, *OECD* Organisation for Economic Co-operation and Development, *PSE* policy, system or environment

Publications were included if they focused on the opinions, attitudes or acceptability of the public, community or population, as well as population subgroups such as policy actors. Only high-income, democratic members of the Organisation for Economic Co-Operation and Development (OECD) across Europe, North America and the Asia–Pacific Region were included to facilitate synthesis and comparisons between studies. Given the influence of democratic freedoms on the expression of public opinion [54], nondemocratic countries were excluded from the review. This approach also updates and builds on the previous review of public opinion about prevention by Diepeveen et al. [32] published in 2013. Included studies were limited from January 2011 to March 2020 to ensure manageability of the scope of the review and avoid duplication of studies included in the previous review.

As the aim of this review was to explore the methods used in peer-reviewed research about public opinion towards primary prevention of NCDs, several types of studies were excluded. Grey literature and unpublished studies were excluded. Risk factors and topics not directly relevant to the major NCD risk factors of interest were excluded: this included injury prevention, mental health and suicide, illicit drugs and breastfeeding. Also excluded from this review were views and attitudes about actual health behaviours, disease or conditions, such as views or attitudes about obesity, unless the major focus of the study was views and attitudes about the policies, strategies or interventions to prevent obesity, for example. A study on the acceptability of an intervention or policy was not included if it was not the major focus of the article, such as a minor part of a broader process evaluation for a programme. We also excluded studies which examined the acceptability of financial-based interventions such as payments for weight loss, as the focus of the review was not on individual-based interventions. The views and opinions of healthcare professionals, clinicians and patients were excluded given the focus of this study was the attitudes of the broader “public” and not only those with a professional or expert interest in health and healthcare.

Some study designs were also excluded, such as formative and experimental studies (those that test the effects or preferences of different messages or frames by different groups and their resulting opinions or attitudes), as this review aimed to analyse descriptive studies of public opinion. We also excluded reviews (including systematic reviews) and meta-analyses to avoid a double-count of original studies.

### Screening process

The first author (A1) completed all database searches, extracted the results and removed duplicates. A1 completed an initial screen of titles and abstracts, removing those outside of the years, countries and risk factors identified. A1 and A2 then screened titles and abstracts using Covidence [55], double-screening 20% to achieve concordance. A1 and A2 completed the full-text screening and agreed on final inclusion of articles in the review.

### Data extraction and analysis

A1 and A2 developed and tested the data extraction tool in Covidence. Included studies were then extracted by A1 from Covidence. The following data were extracted:

- Title of study
- Country or countries of study
- Research type
- Study design
- Chronic disease risk factor or topic group
- Population group
- Setting
- Total number of participants
- Summary of main findings
- Other notes, including details about recruitment, further information about study type and response rate for surveys.

Data about country, study design or method and population were grouped into larger categories agreed upon by authors A1, A2, A5 and A6 for descriptive analysis. Quantitative methods included cross-sectional study designs and cohort studies. Qualitative methods included focus groups, interviews, deliberative processes and some types of media analyses. Mixed methods included those that involve both quantitative and qualitative methods, such as content analysis of news media.

A quality assessment was not conducted given the broad range of methods and study designs included. However, data such as recruitment, sample size/number of participants and survey response rates were collected during the data extraction stage.

## Results

**Fig. 1:**
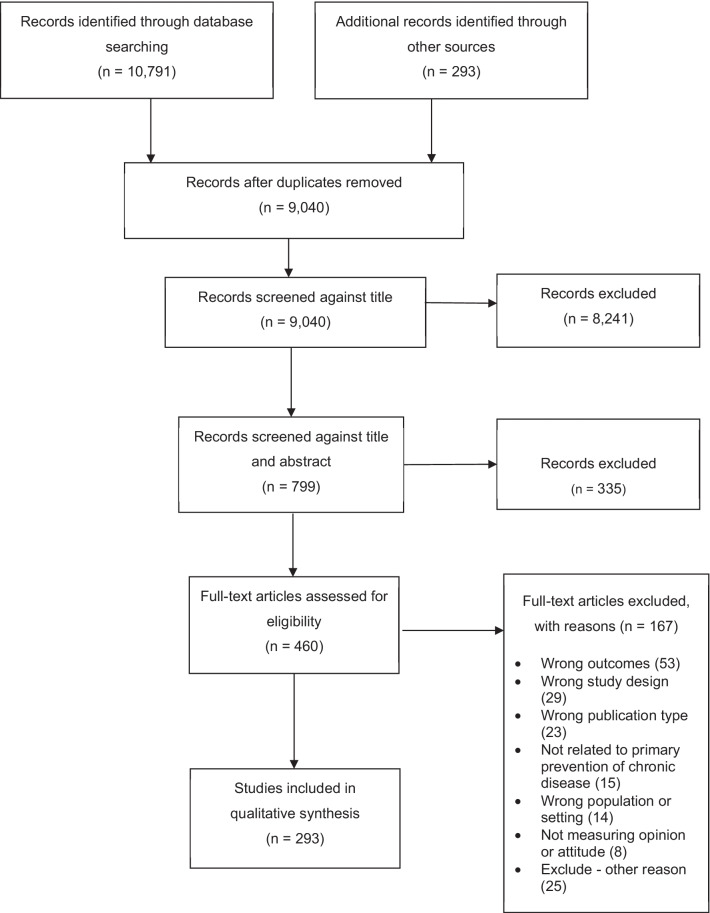
PRISMA diagram

A total of 11,084 titles were identified through database search (*n* = 10,791) and manual search of reference lists of relevant reviews and literature (*n* = 293). Duplicates (*n* = 2044) were removed, leaving 9040 titles which were further screened for relevance, with a further 8241 titles then excluded. A total of 799 abstracts and titles were screened. The full texts of 460 articles were then screened, and of these, 293 articles were included for analysis (Fig. 1 PRISMA diagram).

The full list of included studies with summary of key data extracted can be found in Additional file 3.

### Summary of included studies

Two thirds of the included studies used quantitative methods (*n* = 194, 66%), with a smaller number using qualitative methods (*n* = 60, 20%) or mixed methods (both qualitative and quantitative methods) (*n* = 39, 13%) (Table 2, Methodology and design of included studies).

**Table 2:** Methodology and design of included studies

	Included studies	Proportion of total studies
Quantitative	194	66%
Cross-sectional study—representative sample	129	44%
Cross-sectional study—convenience or purposive sample	42	14%
Cohort study	23	8%
Qualitative	60	20%
Interviews	21	7%
Focus groups	21	7%
Media analysis	8	3%
Deliberative (e.g. citizen’s jury)	5	2%
Document or submission analysis	3	< 1%
Multiple qualitative methods	2	< 1%
Mixed methods	39	13%
Media analysis	17	6%
Multiple quantitative and qualitative methods	12	4%
Cross-sectional survey—convenience sample	7	2%
Document or submission analysis	1	< 1%
Community-based participatory	1	< 1%
Total	293	100%

Quantitative methods included cross-sectional studies of a sample or population, such as surveys of representative (*n* = 129, 44%) or convenience/purposive (*n* = 42, 14%) samples and longitudinal cohort studies (*n* = 23, 8%). Qualitative data collection methods included focus groups (*n* = 21, 7%), interviews (*n* = 21, 7%), qualitative media analysis (*n* = 8, 3%) and deliberative methods (*n* = 5, 2%). Mixed methods included media analysis studies with qualitative and quantitative methods, for example, content analysis (*n* = 17, 6%). Other studies in this category included 12 studies (4%) with multiple study designs incorporating both qualitative and quantitative methods, for example, using focus groups and a survey.

Most studies were conducted at the national level (*n* = 124, 42%), followed by local level (*n* = 85, 29%), state or regional level (*n* = 59, 20%), and international (*n* = 25, 9%). The largest number of studies were conducted in North America (United States or Canada, *n* = 126, 43% of total). Studies from Australia and New Zealand formed a quarter of the studies (*n* = 76, 26%), while studies in other countries were less frequent (United Kingdom and Ireland, *n* = 32, 11%; Europe, *n* = 30, 10%). Nine percent of studies (*n* = 25) were conducted across multiple countries and were deemed “international” studies.

Over half of the studies focused on the general public (*n* = 163, 56%). Fewer studies focused on specific populations, such as policy actors (*n* = 27, 9%), which included government policy-makers, politicians, advocates, lobbyists, academics, retailers and/or industry (*n* = 27, 9%). Other population groups included children, adolescents or young adults (*n* = 19, 6%), smokers or former smokers (*n* = 15, 5%) and university/college students or staff (*n* = 14, 5%). A small number of studies looked at multiple populations and groups (*n* = 15, 5%). Other community groups (*n* = 7, 2%) included public housing residents, Indigenous or culturally and linguistically diverse people and migrant groups.

**Fig. 2:**
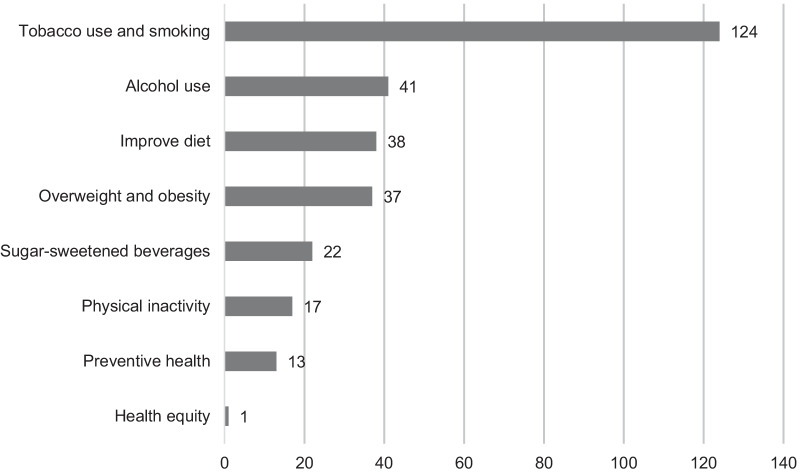
Number of studies by risk factor or topic

Over two fifths of studies (*n* = 124, 42%) were focused on opinions and attitudes regarding prevention of tobacco use and smoking (Fig. 2, Number of studies by risk factor or topic).

Other risk factors or topics included alcohol use (*n* = 41, 14%), improving diet (*n* = 38, 13%) and overweight and obesity (*n* = 37, 13%). A smaller number of studies addressed SSBs (*n* = 22, 8%). Only 17 studies (6%) looked at attitudes about preventive interventions regarding physical inactivity. Thirteen studies (4%) focused on interventions targeting multiple risk factors or the topic of prevention more generally.

### Quantitative methods

#### Cross-sectional study—representative samples

Over half of these studies (*n* = 77, 26%) used a single survey design, that is, conducted once. A smaller number of studies analysed repeat or serial surveys using the results of a single survey wave (*n* = 27, 9%) or by comparing the results of multiple survey waves (*n* = 25, 9%).

##### Single surveys

These studies tended to be of the general public, mostly sampled at a national (country) level (*n* = 42, 14%). Single surveys with regional samples (*n* = 20, 7%) were most often found in larger countries with state jurisdictions, such as the United States or Canada. Examples of these studies included public support for an SSB tax in Kansas, United States [56] and interventions targeting SSBs in Quebec, Canada [57]. Single, local-level representative surveys (*n* = 10, 3%) also tended to be from the United States, such as support for nutrition PSE change strategies in Los Angeles [10].

Tobacco use was a commonly addressed risk factor in cross-sectional studies using a single survey design (*n* = 30, 10%). Half of these (*n* = 15) were conducted in the United States. Surveys were also conducted about increased regulation of tobacco in Australian states [58, 59], New Zealand [60–62] and Scandinavian countries [63]. A small number of single surveys were conducted across multiple countries to compare community attitudes regarding tobacco control in different sociopolitical contexts [64, 65].

Less common risk factors covered by single surveys included improving diet (*n* = 10), alcohol use (*n* = 9), obesity (*n* = 8) and SSBs (*n* = 8). Only seven studies asked about physical inactivity interventions; six of these studies were in North American populations, and were focused on views about active transport measures in the United States [66, 67].

A further seven studies considered views about a broad range of interventions to address chronic disease, including a survey of Canadians about health equity interventions [68] and six surveys asking about preventive health interventions addressing multiple risk factors [69–72].

##### Repeat or serial surveys

Some cross-sectional studies used repeat or serial surveys, with data collected in different “waves” over time. These studies generally used the same survey questions but with different samples each time. In this section we have included studies that analysed both one wave and multiple waves.

Six studies analysed results from SummerStyles (formerly HealthStyles), a market research survey of United States adults run several times a year that collects health-related opinions and attitudes. These studies mainly analysed data from views regarding tobacco control [73–76], though one study was about attitudes regarding urban design to promote physical activity [77] and another about improving fruit and vegetable access [78].

Other examples of major cross-sectional serial surveys include the Eurobarometer survey, a cross-national survey conducted by the European Commission several times per year, including both standard modules and special topic modules in various public policy areas. Each survey wave usually includes over 20,000 participants. In recent years this survey has included questions on attitudes towards tobacco control measures [79–82] and policies to address childhood overweight and obesity [83]. Other international studies had very large samples, such as tens of thousands of participants in multiple countries, in order to compare views about alcohol policies across Europe [84].

Some national-level, long-running serial surveys that tracked public opinions over time were also identified in this review, with studies analysing data from the Social Climate Survey of Tobacco Control in the United States, running since 2000 [85–87]; the National Adult Tobacco Survey, run by the Centers for Disease Control and Prevention in the United States since 2009 [88–91]; and the National Drug Strategy Household Survey in Australia, running since 1995 [92–94]. Other studies used the same survey questionnaire at several points in time to measure changes in attitude and support, for example three national surveys on alcohol control attitudes of 1000 Irish adults in 2002, 2006 and 2010 [95], and a comparison of attitudes about tobacco control across two universities in the United States [96].

Serial surveys of adolescents’ views about prevention policies were also identified, such as tobacco control surveys in the United Kingdom [97], New Zealand [98] and California, United States [99]; and across multiple policy areas in Europe [100].

#### Cross-sectional study—convenience or purposive sample

Some studies used convenience or purposive samples for quantitative cross-sectional surveys of public opinion (*n* = 42, 14%). These were usually to support implementation locally or regionally—most of these studies (*n* = 35, 12%) were conducted in a local or state setting.

Cross-sectional studies using convenience samples were more likely to be surveys of groups or subpopulations, focused on measuring the acceptability of smoke-free settings. For example, eight studies were conducted in a university or college population, seven of which were specifically focused on tobacco control and smoke-free campuses. Other similar studies considered views of employees and managers regarding smoke-free hospitals [101, 102] and public housing residences [103, 104]. Numbers of participants within such settings were much smaller than for other types of cross-sectional surveys, with 1000 or fewer participants in each survey.

Other studies in this category included five studies of policy actors, influencers or stakeholders, including an intercept survey of local retailers’ views about smoke-free shopping streets in Wellington, New Zealand [105]. The other four studies included a survey of political representatives’ support for obesity reduction policies in the United States [106]. These studies again had much smaller numbers of participants (i.e., 200–500 participants) and tended to utilize purposive sampling methods.

#### Cohort study (including sub-studies)

Many prospective or longitudinal cohort studies (*n* = 23, 8%), also known as panel surveys, were identified in this review. This group included both cross-sectional sub-study surveys (at one point in time) within a cohort study (*n* = 15) and ongoing tracking of the cohort over time (*n* = 8). Most cohort studies were in tobacco control (*n* = 19). For example, the Indiana University Smoking Survey [107, 108] is a longitudinal cohort study tracking adolescents’ attitudes about tobacco control over time.

One key type of cohort study identified in this review is the International Tobacco Control (ITC) Policy Evaluation Project [109], a major collaborative effort across 29 countries to measure and track the impact of national-level WHO FCTC policies including advertising bans, labelling, smoke-free legislation and taxation of tobacco products. The ITC study surveys the general public as well as former and current smokers. Twelve studies included in this review came from the ITC study, both across multiple countries as well as country-level specific studies, including the general public in Canada [110], France [111] and New Zealand [112]. Seven of these studies were cross-sectional sub-study surveys drawn from the ITC cohort, including comparative surveys across multiple countries [113, 114].

Of the other 15 cross-sectional sub-study surveys identified, two were drawn from existing prospective cohort studies in Australia. One sample among parents participating in a birth cohort study surveyed them about policy preferences for preventing childhood obesity [115]. Another survey looked at support for active transport policies amongst a cohort of cyclists [116]. The other sub-studies were drawn from the NutriNet-Santé cohort study in France [117] and the International Food Policy study looking at support for healthy food policies and diets across five countries [118].

### Qualitative methods

#### Focus groups

The focus group studies (*n* = 21, 7%) were mostly recruited from and conducted within local settings, exploring a range of prevention strategies targeting alcohol use (*n* = 6), tobacco use (*n* = 5) and improving diet (*n* = 4). Several studies were identified that explored specific interventions with a higher level of public contestability, such as alcohol taxation and pricing [119–122] and public attitudes towards preventive pricing policies [123].

While recruiting from the general public was a common source for focus groups (*n* = 12), focus groups studies were also used with specific groups such as adolescents (*n* = 5)—for example, understanding how adolescents in the United Kingdom felt about healthier food in school canteens [124] and adolescent views regarding SSB taxes in the United States [125]. This was also the case for those studies that explored more marginalized socioeconomic groups, such as those regarding obesity prevention [126] and promoting fruit and vegetable access [127].

Focus groups were used in specific settings such as workplaces to understand employees’ perceptions of workplace-based interventions to promote physical activity [128] and university students’ attitudes regarding campus alcohol policies [129, 130].

The total number of participants varied across the included studies, from 24 participants in one group to 218 across 28 focus groups.

#### Interviews

Interviews were another qualitative design used (*n* = 21, 7%). Interviews were commonly used to explore the opinions and attitudes of policy actors (*n* = 16), mainly regarding obesity prevention and tobacco control policy measures. Specific policy or research areas were explored using semi-structured and in-depth interviews, for example about alcohol regulation and taxation in Australia [131] and reducing tobacco availability in New Zealand [132]. Some studies also interviewed policy actors about food-related taxes and subsidies [133] and nutrition labelling [134] in New Zealand and childhood obesity prevention in Spain [135]. One study looked at the differences in views about obesity prevention priorities and strategies among 20 politicians in one state of the United States compared to advocates’ views [136].

Four studies were identified that specifically focused on understanding the opinions and attitudes of retailers and industry. Three of these studies were about tobacco control interventions, such as smoke-free pubs and bars [137] and tobacco “endgame” strategies in New Zealand [138, 139]. The remaining study included interviews with recreation centre managers regarding pricing interventions to promote healthier food choices [140].

Some studies recruited from specific community groups, such as interviews with more marginalized or less studied communities—for example, interviewing children about restricting junk food marketing in New Zealand [141] or interviewing adolescents about nutrition interventions in disadvantaged communities in Australia [142], and interviewing disadvantaged and low-income smokers about increased tobacco control regulation [143, 144].

#### Media analysis—qualitative

Qualitative media analysis studies (*n* = 8, 3%) involved news media analysis (*n* = 3), analysis of reader comments on news stories (*n* = 3) and social media analysis (*n* = 2). These studies were from the United States (*n* = 3), United Kingdom (*n* = 3), Australia (*n* = 1) or multiple countries (*n* = 1).

Three studies analysed English-language print and/or online news media reporting on obesity and tobacco control. One study used framing analysis to look at how newspapers over a 10-year period reported on childhood obesity in the United States [145]. Another study reviewed news media articles on solutions to address childhood obesity over a 20-year period, comparing and contrasting reporting across three countries, the United States, Canada and the United Kingdom [146]. At a state level in the United States, Kuiper et al. [147] analysed news coverage of smoke-free laws in Michigan.

Two studies used social media analysis to measure and understand public opinion about a specific preventive topic. Astill Wright et al. [148] analysed tweets about minimum unit pricing of alcohol in Scotland. Feng et al. [149] also analysed Twitter regarding proposed new tobacco taxation levels in California.

Three of the media analysis studies examined reader commentary on news media articles. One study analysed over 3600 comments on 83 news media stories in Australia focused on obesity [150]. Another study analysed 1645 reader comments on articles about SSB taxation in the United Kingdom [151].

#### Deliberative processes and citizens’ juries

A small number of qualitative studies (*n* = 5, 2%) used a deliberative process, such as a citizens’ jury, to study public opinion and attitudes. All studies were conducted in Australia at a local or state level using a range of recruitment methods, such as purposive sampling, market research recruitment or recruitment from an existing survey population.

Four of these studies recruited participants from the general population, using 13–20 participants per study. Two were focused on improving diet, such as exploring and understanding attitudes about food policy [152], and the regulation of fast food through a corporate health impact assessment [153]. Another study used a citizens’ jury design to understand whether the government should tax SSBs [154]. The other study looked at community attitudes regarding regulations and laws for childhood obesity prevention [155].

Only one deliberative study was conducted with a specific population. Street et al. [156] ran a community deliberative forum in an Australian town with a local Aboriginal community, exploring what preventive areas and programmes government should invest in to support Aboriginal youth well-being, exploring policy options as well as broader issues such as structural racism and socioeconomic disadvantage.

#### Document or submission analysis

Qualitative studies using document or submission analysis (*n* = 3, 1%) were analyses of parliamentary proceedings and submissions made by stakeholders or members of the general public regarding interventions for alcohol use and diet.

One study analysed industry submissions to a parliamentary inquiry on alcohol, focusing on identifying the tactics used by the alcohol industry to create barriers to preventive alcohol policy in Australia [157]. A similar study was conducted in New Zealand, comparing submissions from the general public, advocacy groups and alcohol industry to explore how different groups responded to a proposal to increase the alcohol purchasing age [158]. Another study looked at Australian politicians’ presentation of public health nutrition issues, using framing analysis of parliamentary transcripts to understand their views on the causes of and solutions to childhood obesity and junk food marketing to children [159].

#### Multiple qualitative methods

Two studies used a combination of study designs with qualitative methods. Katikireddi and Hilton [160] analysed news media, policy submissions and in-depth interviews to compare the ways in which different policy actors influenced public opinion on the topic of alcohol minimum unit pricing in Scotland. The other study in this category included interviews with policy actors and stakeholders in New Zealand (including the tobacco industry) alongside focus groups with smokers, looking at support for low-nicotine-content cigarettes [161].

### Mixed methods

#### Media analysis

Some media analysis studies involved both quantitative and qualitative methods (*n* = 17, 6%) such as content analysis of English-language news media articles (*n* = 15), social media (*n* = 1) and reader commentary on news media (*n* = 1).

Six studies analysed news media coverage of tobacco control measures, such as smoke-free laws in Canada or the United States [162, 163] and bans in the United Kingdom on smoking in cars carrying children [164, 165]. SSB taxation was another popular topic for mixed-method media content analysis during this period, with four studies covering this issue by analysing stakeholders’ views in news media in the United Kingdom [166–168] and in the United States [169]. In terms of alcohol use, two studies conducted content analysis of United Kingdom newspapers to explore how alcohol minimum unit pricing was represented in the media by different groups [170, 171]; a similar study from Australia examined news media coverage of alcohol advertising restrictions [172]. Only one study was identified that used both quantitative and qualitative media analysis to look at news reporting of views about solutions to childhood obesity [173].

Two studies included in this category conducted content analysis of different media platforms. One study looked at tweets on a United States school meals policy, using opinion-mining techniques and content analysis [174]. Freeman [175] analysed 117 online news items (including opinion polls) and reader comments on Australian news media platforms to understand support and opposition to tobacco plain packaging prior to implementation.

#### Multiple quantitative and qualitative methods

Multiple designs or methods (*n* = 12, 4%) encompassed the use of quantitative and qualitative methods, for example conducting a quantitative cross-sectional study as well as using qualitative methods such as focus groups and/or interviews (*n* = 10).

Some studies used qualitative methods as formative research to inform subsequent cross-sectional survey design. Grunseit et al. [176] conducted focus groups with members of the general public regarding views and attitudes about preventive health in Australia, which then informed the development of a national cross-sectional survey about prevention. Booth et al. [177] also used this approach with a purposive (rather than representative) sample; they interviewed school policy actors in the United Kingdom about childhood obesity prevention, with the results informing a cross-sectional survey for a wider group of stakeholders.

Other studies conducted the quantitative and qualitative elements alongside one another. One study from Australia conducted an online survey about strategies to promote public transport use, with “nested” focus groups and interviews [178]. A similar study in the United Kingdom conducted a representative survey of adults regarding views on preventive alcohol policies, from which focus group participants were recruited to explore the topic in greater depth [179]. Other mixed-methods studies conducted in specific settings (e.g., schools or universities) used a similar methodological design (survey plus focus group and/or interviews) to look at acceptability of alcohol measures in Spain [180] or tobacco control in the Netherlands [181].

A small number of studies used other mixed methods, for example conducting document analysis of legislative bills about calorie menu labelling in California in conjunction with news media content analysis to understand how different policy actors shaped the public debate over time [182]. Mah et al. [183] also looked at calorie menu labelling in Canada, using results from a public survey and comparing these with the findings from a survey and policy consultation with hospitality industry stakeholders. Another study recruited 95 members of the general public in New York, United States, to explore views about what communities could do to prevent childhood obesity; the authors first conducted a survey, used Q methodology (to sort statements of participants based on their agreement, views and feelings), followed by structured interviews [184].

#### Cross-sectional survey

A small number of studies (*n* = 7, 2%) utilized cross-sectional surveys that had both closed- and open-ended responses, with thematic or content analysis often used to analyse the open-ended responses; as such, these have been included as “mixed methods”. Over half of these studies were with multiple groups or a specific population at the local level, such as exploring views and attitudes about a health-promoting setting—for example, surveys with university students and staff regarding SSB regulation on campus [185], employee attitudes about a smoke-free hospital campus [186] and views about bike-sharing infrastructure in a Swedish city [187].

#### Other study designs

Other mixed methods were used in a small number of studies (*n* = 3, 1%). These included a Delphi study of policy actors and stakeholders regarding views on physical activity policy measures in the Netherlands [188], a community-based participatory study with policy actors involving concept mapping to promote physical activity in local communities across the United States [189] and a content analysis of public comments in the United States regarding changes to public health nutrition standards [190].

## Discussion

This review is the first to identify and synthesize the methods and study designs used to describe the acceptability of preventive measures and policies to address lifestyle-related chronic disease in high-income, democratic countries across Europe, North America and the Asia–Pacific region.

We identified four major outcomes from this review that have implications for future research directions. First, quantitative cross-sectional study designs are the predominant way in which public opinion about NCD prevention is measured and understood. Second, while qualitative and mixed methods are used less frequently, they may provide an important value add for understanding public opinion which could assist implementation of preventive policies. Third, tobacco control forms a significant portion of the literature, which may have facilitated or contributed to improved policy implementation, with flow-on effects for improving health outcomes. Finally, only a small number of studies considered public opinion about topics such as physical inactivity, indicating a data gap for this area of prevention. Below we take each of these findings and explore their implications for future research.

### Cross-sectional studies are the predominant method for public opinion data

We found that the literature published during the time frame of inclusion relies heavily on quantitative methods, particularly cross-sectional study designs. The reliance on such study designs has implications for the way in which researchers and policy-makers conceptualize and collect data on public opinion, which then affects how we understand this phenomenon. While we did not conduct a quality assessment of all the studies, we make some general comments about the possible challenges in using such methods and study designs.

In public opinion research, cross-sectional studies are the most commonly used method, aiming to provide a “snapshot” of the public’s attitudes at a specific point in time [191]. Most public opinion surveys about prevention identified in this review used representative, probability or random sampling techniques, including recruiting from market research groups and weighting results based on the demographic distribution of the studied population, usually at a national (country) level. Nonresponse bias can arise in terms of study selection, particularly from telephone-based surveys, which can influence the reliability and accuracy of the results [192]. Generally, the lower the response rate the more doubt there could be about the representativeness of the population and their responses [193]. As such, the reported opinions of many of these studies may not be truly representative of the population, though we note that surveys in this review did use larger sample sizes to account for uncertainty, and generally recorded response rates as part of their methods and/or limitations. However, these surveys may still reflect a selective view of what the “majority” thinks, with criticism that survey research relies on capturing a “narrow dimension of public attitudes” [194].

There is also the possible issue of social desirability bias, where survey participants respond based on perceptions about the desirability of certain behaviours over others [195]. This could apply to preventive interventions about behaviours or conditions that are stigmatized, such as smoking or obesity. For example, Knox et al. [196] found some evidence of social desirability bias in a study of United States adults regarding SSB consumption and perceptions about the health and economic benefits of SSB taxation. It is possible that the surveys included in this review experienced some social desirability bias, though equally that could apply to qualitative and other methods that involve discussing topics in front of others (such as focus groups).

Other challenges with relying on cross-sectional survey data for public opinion about prevention include measurement comparability. There is no one standardized approach to measuring people’s opinions either individually or collectively [191], though some studies such as serial or repeated surveys use the same questions over time, and market research companies may conduct pretesting strategies to validate the survey instrument. Regardless, there are still issues in terms of reliability and comparability if different surveys are using different instruments. These issues can also apply between different survey modalities, such as telephone-based surveys versus internet surveys [197].

While well-run cross-sectional surveys are useful, the results from these studies represent one type of evidence or representation of public opinion and acceptability, rather than the only type. This point has been noted in other studies that incorporate diverse forms of quantitative and qualitative evidence in defining the “acceptability” of interventions [198].

### Qualitative and mixed methods have a value add for this area of research

Our findings suggest that while qualitative and mixed-methods research on opinions and attitudes were much less commonly used than quantitative methods, they may play an important and complementary role. Although quantitative methods may help to explain the “what” people think about a certain question, they can’t tell “why” people may hold those views—the beliefs, values, ideologies and ethical positions of participants. Qualitative and mixed methods can help build a richer picture of public opinion, explore why people hold opinions and attitudes, and look at broader assumptions, values, and beliefs about health, government and responsibility [176].

We found examples of studies that used different methodological approaches in a complementary way. Li et al. [179] used mixed methods (cross-sectional survey and focus groups) to explore attitudes about alcohol control policies in the United Kingdom. Somerville et al. [123] noted the importance of using qualitative methodology, such as focus groups, to complement quantitative methods in acceptability studies in public health. These methods can help to illustrate that people’s views on policies develop through interactions with others. In other words, expression of support (or disagreement) with a policy or intervention is not static but part of a dynamic process of opinion formation [194].

We also identified some novel and innovative methodological approaches in the qualitative literature. Social media analysis of Twitter users provided insight into the salience of a specific and contested preventive policy, such as minimum unit pricing of alcohol in the United Kingdom [148]. Social media provides one avenue for the public to comment on public health interventions outside of traditional polling methods, and could be particularly useful when studying examples of policy implementation failure, such as in the case of California’s attempt to increase tobacco taxation [149]. However, there is caution about how much these platforms are representative of the “public” or community at large. This is particularly important given that unhealthy industry groups may use social media platforms such as Twitter as part of their lobbying strategy to influence public and political opinion [199].

Other innovative methods we identified that provide a rich body of evidence included deliberative methods, which can generate ideas on policy solutions from participants as well as observing and understanding the process of change in opinions and attitudes [153], and “Q methodology”, which was used to find common ground across the general public for childhood obesity prevention and to identify the least contested strategies which could be acceptable [184].

There is moreover a vital role for qualitative methods in terms of giving “voice” to underserved, vulnerable or marginalized groups that may be left out of larger national quantitative surveys. Examples include focus groups with children and adolescents, particularly in disadvantaged areas [142], in-depth interviews of low-income smokers [143] and a deliberative dialogue with an Aboriginal community regarding youth well-being [156]. Qualitative research has a powerful part to play in addressing inequities in research, particularly in public opinion research which has tended to empirically focus on what the “majority” think through public polls and surveys. However, we do note the limitations of study designs like focus groups, which can have a different kind of “majority opinion” effect on participation; this has been identified in other reviews of the literature [33].

### Learning from tobacco control about public opinion and acceptability

This review identified that tobacco control has a much larger published evidence base about attitudes and opinions compared to the other risk factors included in this study. It is possible such an extensive evidence base has likely facilitated or contributed to policy implementation of tobacco control strategies in the countries included in this review.

Most tobacco control studies were predominantly annual cross-sectional surveys with large samples across multiple countries. Multi-country surveys are useful for being able to place public opinion about prevention within a specific sociopolitical and policy context—for example, demonstrating that increased tobacco control implementation at a country level is positively related to the social denormalization of tobacco and linked with increased support for existing or proposed tobacco control measures [81]. A similar relationship exists in terms of support for tobacco “endgame” strategies and the advanced implementation of country-level tobacco control policies [64]. Some of these surveys were also conducted in multiple “waves” which could detect temporal changes. This type of evidence could be persuasive for political leaders and policy-makers who are unsure about implementing new preventive interventions and policies, but we note that such long-running international surveys are both expensive and time-consuming.

In tobacco control, we also found cohort studies for tracking public opinion and attitudes. The benefit of these studies is that they can detect associations between exposure—for example, adolescents’ exposure to increasing implementation of tobacco control measures—and changes in views and attitudes [107, 108, 200]. Studies like the ITC Study have contributed enormously to knowledge and evidence about the acceptability of tobacco control to inform policy implementation. It is therefore promising to see similar studies developed in other areas of prevention, such as the International Food Policy Study [118]. Such studies are costly but valuable sources of evidence, as public opinion data from these kinds of study designs may help to distinguish between age, period or cohort effects [201, 202]. This is an important methodological question for public opinion research, given the challenges of measurement error and bias and the reflection of past views in cross-sectional surveys [203]. Given the large numbers of existing longitudinal cohort studies tracking preventive health behaviours, researchers may wish to consider attitudinal questions and measures as part of data collection, especially those in other areas of prevention outside of tobacco control.

In addition, there were many local-level convenience or purposive surveys and setting-specific surveys about tobacco control. While these may be a less rigorous form of evidence compared to large nationally representative surveys, they are still useful (and cheaper) at providing a snapshot of community views, which may facilitate local-level policy implementation, such as prior to introducing policies or guidelines, or “temperature-testing” of new interventions. This could explain why many of the included studies using this type of method were focused on smoke-free regulations in specific settings like universities, public places or hospitals, with findings that could be useful for improved health policy implementation.

### Public opinion about physical inactivity—an evidence gap?

Our review demonstrates that physical inactivity was rarely the focus of studies looking at opinions and attitudes. This finding echoes that of the review by Diepeveen et al. [32], which identified a small number of studies looking at people’s views and attitudes about prevention policies and interventions relating to physical inactivity. This gap could be for several reasons.

While there has been increased focus on physical inactivity as a major risk factor for chronic disease, particularly with the development of international strategic frameworks such as the global action plan on physical activity [204], the findings from our review suggest a major evidence gap in terms of understanding people’s acceptability and opinions about interventions. Generating such evidence could assist governments in implementing such frameworks and recommended policies. Monitoring policy support is one type of “macro-level determinant” for physical activity that needs to be included as part of population surveillance [205].

Some have commented that physical inactivity is the so-called Cinderella risk factor in chronic disease prevention, receiving little policy attention and focus compared to other more prominent risk factors such as tobacco [206]. Other empirical research has found that physical inactivity is reported far less frequently in the news media than tobacco or obesity [207]. Our review results indicate that this lack of attention to physical inactivity also seems to apply to assessing public opinion and community attitudes.

Finally, we do note that many interventions targeting physical inactivity are multisectoral in nature; that is, they occur outside the health sector, such as transport interventions, congestion charging or pricing, and urban planning interventions. It is possible that studies reporting on public opinions about such interventions are not published in the public health literature and therefore were not identified in our search strategy.

### Strengths and limitations

The strengths of this review include the use of systematic search terms and processes across four databases along with manual searching of previous and related reviews. While double-screening and reviewing were not used, the first two authors achieved high concordance with 20% of studies double-screened.

Some limitations of this review include the lack of agreed terminology for terms such as “opinions”, “attitudes” and “acceptability”, which makes a systematic search for this literature challenging. Similarly, “prevention” can be used in many different contexts. To address this, the reviewers used an inclusive search strategy, which did result in a large number of results that had to be screened. However, some studies may still have been missed. As such this is not a comprehensive systematic review. A full quality assessment of each study was also not performed during the data extraction stage. While some information was gathered (such as sample size and response rates), quality assessment of methods was not possible due to the heterogeneity of studies. This means that methods have been discussed in terms of their general quality. Future research might consider a systematic analysis of study designs and outcomes, though this would be challenging given the significant heterogeneity of methods and other issues that we have outlined in this review.

Another limitation relates to possible publication bias. Only studies published in peer-reviewed journals in the English language were included. This excludes those studies which are not published, including the grey literature, and it also excludes non-English-language publications. Therefore, the patterns of methods in studies in those areas could differ from those reported here. It is important to note that governments and organizations often use market research companies to research how current and hypothetical policies are accepted by the population, but that these are not normally publicly available. Future research could include reviewing the grey literature and explore using access to information laws to obtain the unpublished, market research studies.

Finally, we note that our review was restricted to high-income, democratic countries across Europe, North America and the Asia–Pacific region. Future research could look at replicating this style of review and considering evidence from other countries and regions of the world to compare methods, study designs and areas of study. Furthermore, such research could highlight the effect of different political, social and economic contexts regarding NCD prevention policy and systems change.

## Conclusion

This review has provided an update to previous reviews in this area of research in high-income, democratic countries across Europe, North America and the Asia–Pacific. The majority of studies in this review used quantitative methods, with few using qualitative methods. We also found that tobacco control was the dominant topic investigated in public opinion literature.

These findings demonstrate that opportunities exist to investigate the role of public opinion not only in other prevention topic areas, but also through the use of qualitative and mixed methods to provide more nuanced insights which may facilitate policy implementation of strategies to prevent NCDs. Public opinion and acceptability of prevention is an important feature of effective implementation of preventive strategies; however, there are a variety of ways in which it can be measured and understood. This review has identified some gaps and opportunities for future research which may assist in convincing decision-makers and the broader public of the need to act on NCDs, and to prioritize and implement more upstream preventive strategies and policies.

## Supplementary Information

**Additional file 1:** PRISMA Checklist.

**Additional file 2:** Search terms and results.

**Additional file 3:** Studies included in review.

## Data Availability

Relevant data and materials are provided under additional files. The datasets used and/or analysed during the current study are available from the corresponding author on reasonable request.
