# Supplementary material for: Measuring public opinion and acceptability of prevention policies: an integrative review and narrative synthesis of methods
Source: Health Res Policy Syst. 2022 Mar 4;20:26. doi: 10.1186/s12961-022-00829-y (PMC8895540; doi:10.1186/s12961-022-00829-y)
Supplement: Supplementary file 2 — Additional file 2: Search terms and results. [file 12961_2022_829_MOESM2_ESM.docx]

**Supplementary material S2**. *Search terms and results*

**Scopus (4367 results)**

*28 February 2020*

( ( attitud* OR opinion* OR belief* OR view* OR support OR acceptability ) W/2 ( community OR public OR population OR adult* OR child* OR adolescent* OR "young adult" ) ) AND

( prevent* OR policy OR policies OR ( ( health OR fiscal OR nutrition* OR alcohol OR smok* ) W/2 ( policies OR policy ) ) OR tax* OR regulat* OR government OR "health promotion" OR ( ( food OR product ) W/2 label* ) OR planning ) AND

( diet* OR nutrition* OR food OR "health behavio?r*" OR lifestyle* OR "physical activity" OR "sedentary behavio?r" OR "active travel" OR transport OR cycli* OR walk* OR "built environment" OR tobacco OR smok* OR cigarette* OR alcohol* OR obes* OR overweight ) ) AND

PUBYEAR > 2010

**Ovid Medline (3214 results)**

*28 February 2020*

| **#** | **Search Statement** | **Results** |
| --- | --- | --- |
| 1 | Public Opinion/ | 18406 |
| 2 | attitude/ or attitude to health/ | 128992 |
| 3 | (attitude* or opinion* or views or viewpoint* or belief* or acceptability).tw,kf. | 428211 |
| 4 | 1 or 2 or 3 | 522500 |
| 5 | policy/ or fiscal policy/ or smoke-free policy/ or social control policies/ or public policy/ or health policy/ or nutrition policy/ | 107851 |
| 6 | social control, formal/ or government regulation/ or social control, informal/ or social planning/ or city planning/ or environment design/ or urban renewal/ | 42315 |
| 7 | government regulation/ or legislation as topic/ or legislation, food/ | 37901 |
| 8 | economics/ or economics, behavioral/ or taxes/ | 33984 |
| 9 | (policy or policies or legislation or regulat* or tax or taxes or taxation or governance or paternal*).tw,kf. | 2120306 |
| 10 | ((food or nutrition*) and label*).tw,kf. | 15623 |
| 11 | 5 or 6 or 7 or 8 or 9 or 10 | 2245361 |
| 12 | 4 and 11 | 52947 |
| 13 | Diet/ or "Diet, Food, and Nutrition"/ or Healthy Diet/ | 159131 |
| 14 | exp healthy lifestyle/ or sedentary behavior/ | 13981 |
| 15 | smoking cessation/ or smoking reduction/ or "tobacco use cessation"/ | 28934 |
| 16 | obesity/ or pediatric obesity/ | 183822 |
| 17 | (diet* or nutrition* or overweight or obes* or "sedentary behavio?r*" or "physical activity" or "physical inactivity").tw,kf. | 1064458 |
| 18 | ((healthy or sedentary) adj3 lifestyle*).tw,kf. | 11952 |
| 19 | ((Smok* or cigarette*) adj3 (stop* or cease* or cessation or quit*)).tw,kf. | 34209 |
| 20 | drinking behavior/ or alcohol drinking/ | 71940 |
| 21 | (alcohol* or "alcohol control").tw,kf. | 330762 |
| 22 | bicycling/ or walking/ | 41835 |
| 23 | 13 or 14 or 15 or 16 or 17 or 18 or 19 or 20 or 21 or 22 | 1510853 |
| 24 | 4 and 11 and 23 | 5791 |
| 25 | limit 24 to (english language and yr="2011 - 2020") | 3214 |

**EMBASE (1877 results)**

*13 March 2020*

| **#** | **Search Statement** | **Results** |
| --- | --- | --- |
| 1 | public opinion/ | 18035 |
| 2 | attitude/ or attitude to health/ | 174859 |
| 3 | health belief/ | 10806 |
| 4 | social belief/ | 2627 |
| 5 | (attitud* or opinion* or view* or viewpoint* or belief* or acceptability or support or preference).mp. | 2682127 |
| 6 | 1 or 2 or 3 or 4 or 5 | 2682127 |
| 7 | (community or population or public).mp. | 3404559 |
| 8 | 6 and 7 | 501720 |
| 9 | public policy/ or nutrition policy/ or health care policy/ or fiscal policy/ or policy/ or organizational policy/ | 285000 |
| 10 | smoking ban/ or smoking regulation/ | 2714 |
| 11 | government regulation/ | 26431 |
| 12 | social control/ | 15302 |
| 13 | social norm/ | 2874 |
| 14 | city planning/ or environmental planning/ or planning/ | 53724 |
| 15 | tax/ | 15707 |
| 16 | economics/ or behavioral economics/ or health economics/ | 273066 |
| 17 | food packaging/ | 10090 |
| 18 | (policy or policies or legislation or regulat* or tax* or governance).mp. | 3385419 |
| 19 | ((food or nutrition* or alcohol) and label*).mp. | 42063 |
| 20 | diet/ or obesogenic diet/ or Western diet/ or healthy diet/ or unhealthy diet/ | 264601 |
| 21 | fast food/ or junk food/ or food/ | 99743 |
| 22 | nutrition/ | 119478 |
| 23 | (diet* or nutrition* or food).mp. | 1959752 |
| 24 | childhood obesity/ or obesity/ | 437969 |
| 25 | body mass/ | 423272 |
| 26 | body weight/ | 321312 |
| 27 | (overweight or obes*).mp. | 586417 |
| 28 | physical activity/ or physical inactivity/ | 156628 |
| 29 | sedentary lifestyle/ or exercise/ | 306595 |
| 30 | walking/ or walkability/ | 69599 |
| 31 | cycling/ or bikeability/ | 11996 |
| 32 | "traffic and transport"/ | 15640 |
| 33 | built environment/ | 482 |
| 34 | (physical activity or physical inactivity or active travel).mp. | 211698 |
| 35 | alcohol consumption/ | 125363 |
| 36 | drinking behavior/ | 50034 |
| 37 | alcoholic beverage/ | 8224 |
| 38 | alcohol control.mp. | 651 |
| 39 | tobacco/ or tobacco consumption/ or "tobacco use"/ | 60033 |
| 40 | smoking/ or smoking regulation/ or cigarette smoking/ or smoking cessation/ or smoking prevention/ or smoking reduction/ | 394272 |
| 41 | (smok* or cigarette*).mp. | 536457 |
| 42 | healthy lifestyle/ or lifestyle/ | 114801 |
| 43 | health behavior/ | 64969 |
| 44 | ((healthy or sedentary) adj3 lifestyle*).mp. | 29780 |
| 45 | 9 or 10 or 11 or 12 or 13 or 14 or 15 or 16 or 17 or 18 or 19 | 3700770 |
| 46 | 20 or 21 or 22 or 23 or 24 or 25 or 26 or 27 or 28 or 29 or 30 or 31 or 33 or 34 or 35 or 36 or 37 or 38 or 39 or 40 or 41 or 42 or 44 | 3597792 |
| 47 | ((attitud* or prefer* or opinion* or perception* or belief* or view* or support) adj2 (community or public or population or adult* or child* or adolescent* or "young adult")).mp. | 67822 |
| 48 | 45 and 46 and 47 | 3064 |
| 49 | limit 48 to (english language and yr="2011 - 2020") | 1877 |

**Cinahl (EBSCO) (1,333 results)**

*13 March 2020*

| [Search ID#](javascript:__doPostBack('ctl00$ctl00$FindField$FindField$historyControl$ReorderHistoryLink','')) | **Search Terms** | **Search Options** | **Actions** |
| --- | --- | --- | --- |
| S27 | S9 AND S10 AND S23 | **Limiters** - English Language; Published Date: 20110101-20191231  **Expanders** - Apply equivalent subjects  **Search modes** - Boolean/Phrase | [**View Results**](javascript:__doPostBack('ctl00$ctl00$FindField$FindField$historyControl$HistoryRepeater$ctl00$linkResults','')) (1,333)  [**View Details**](javascript:showShDetails(%22ctl00_ctl00_FindField_FindField_historyControl_ctrlPopup%22,%20%22S27%22);)  [**Edit**](http://web.a.ebscohost.com.ezproxy1.library.usyd.edu.au/Legacy/Views/UserControls/EHOST/) |
| S26 | S9 AND S10 AND S23 | **Limiters** - Published Date: 20110101-20191231  **Expanders** - Apply equivalent subjects  **Search modes** - Boolean/Phrase | [**View Results**](javascript:__doPostBack('ctl00$ctl00$FindField$FindField$historyControl$HistoryRepeater$ctl01$linkResults','')) (1,353)  [**View Details**](javascript:showShDetails(%22ctl00_ctl00_FindField_FindField_historyControl_ctrlPopup%22,%20%22S26%22);)  [**Edit**](http://web.a.ebscohost.com.ezproxy1.library.usyd.edu.au/Legacy/Views/UserControls/EHOST/) |
| S25 | S9 AND S10 AND S23 | **Limiters** - Published Date: 19810101-20191231  **Expanders** - Apply equivalent subjects  **Search modes** - Boolean/Phrase | [**View Results**](javascript:__doPostBack('ctl00$ctl00$FindField$FindField$historyControl$HistoryRepeater$ctl02$linkResults','')) (2,158)  [**View Details**](javascript:showShDetails(%22ctl00_ctl00_FindField_FindField_historyControl_ctrlPopup%22,%20%22S25%22);)  [**Edit**](http://web.a.ebscohost.com.ezproxy1.library.usyd.edu.au/Legacy/Views/UserControls/EHOST/) |
| S24 | S9 AND S10 AND S23 | **Expanders** - Apply equivalent subjects  **Search modes** - Boolean/Phrase | [**View Results**](javascript:__doPostBack('ctl00$ctl00$FindField$FindField$historyControl$HistoryRepeater$ctl03$linkResults','')) (2,160)  [**View Details**](javascript:showShDetails(%22ctl00_ctl00_FindField_FindField_historyControl_ctrlPopup%22,%20%22S24%22);)  [**Edit**](http://web.a.ebscohost.com.ezproxy1.library.usyd.edu.au/Legacy/Views/UserControls/EHOST/) |
| S23 | S11 OR S12 OR S13 OR S14 OR S15 OR S16 OR S17 OR S18 OR S19 OR S20 OR S21 OR S22 | **Expanders** - Apply equivalent subjects  **Search modes** - Boolean/Phrase | [**View Results**](javascript:__doPostBack('ctl00$ctl00$FindField$FindField$historyControl$HistoryRepeater$ctl04$linkResults','')) (663,378)  [**View Details**](javascript:showShDetails(%22ctl00_ctl00_FindField_FindField_historyControl_ctrlPopup%22,%20%22S23%22);)  [**Edit**](http://web.a.ebscohost.com.ezproxy1.library.usyd.edu.au/Legacy/Views/UserControls/EHOST/) |
| S22 | (MH "Life Style+") | **Expanders** - Apply equivalent subjects  **Search modes** - Boolean/Phrase | [**View Results**](javascript:__doPostBack('ctl00$ctl00$FindField$FindField$historyControl$HistoryRepeater$ctl05$linkResults','')) (201,662)  [**View Details**](javascript:showShDetails(%22ctl00_ctl00_FindField_FindField_historyControl_ctrlPopup%22,%20%22S22%22);)  [**Edit**](http://web.a.ebscohost.com.ezproxy1.library.usyd.edu.au/Legacy/Views/UserControls/EHOST/) |
| S21 | (MH "Smoking+") OR (MH "Smoking Cessation") | **Expanders** - Apply equivalent subjects  **Search modes** - Boolean/Phrase | [**View Results**](javascript:__doPostBack('ctl00$ctl00$FindField$FindField$historyControl$HistoryRepeater$ctl06$linkResults','')) (64,618)  [**View Details**](javascript:showShDetails(%22ctl00_ctl00_FindField_FindField_historyControl_ctrlPopup%22,%20%22S21%22);)  [**Edit**](http://web.a.ebscohost.com.ezproxy1.library.usyd.edu.au/Legacy/Views/UserControls/EHOST/) |
| S20 | (MH "Tobacco Products+") | **Expanders** - Apply equivalent subjects  **Search modes** - Boolean/Phrase | [**View Results**](javascript:__doPostBack('ctl00$ctl00$FindField$FindField$historyControl$HistoryRepeater$ctl07$linkResults','')) (3,947)  [**View Details**](javascript:showShDetails(%22ctl00_ctl00_FindField_FindField_historyControl_ctrlPopup%22,%20%22S20%22);)  [**Edit**](http://web.a.ebscohost.com.ezproxy1.library.usyd.edu.au/Legacy/Views/UserControls/EHOST/) |
| S19 | (MH "Alcohol Drinking+") | **Expanders** - Apply equivalent subjects  **Search modes** - Boolean/Phrase | [**View Results**](javascript:__doPostBack('ctl00$ctl00$FindField$FindField$historyControl$HistoryRepeater$ctl08$linkResults','')) (28,188)  [**View Details**](javascript:showShDetails(%22ctl00_ctl00_FindField_FindField_historyControl_ctrlPopup%22,%20%22S19%22);)  [**Edit**](http://web.a.ebscohost.com.ezproxy1.library.usyd.edu.au/Legacy/Views/UserControls/EHOST/) |
| S18 | (MH "Built Environment") OR (MH "Obesogenic Environment") | **Expanders** - Apply equivalent subjects  **Search modes** - Boolean/Phrase | [**View Results**](javascript:__doPostBack('ctl00$ctl00$FindField$FindField$historyControl$HistoryRepeater$ctl09$linkResults','')) (134)  [**View Details**](javascript:showShDetails(%22ctl00_ctl00_FindField_FindField_historyControl_ctrlPopup%22,%20%22S18%22);)  [**Edit**](http://web.a.ebscohost.com.ezproxy1.library.usyd.edu.au/Legacy/Views/UserControls/EHOST/) |
| S17 | (MH "Walking+") | **Expanders** - Apply equivalent subjects  **Search modes** - Boolean/Phrase | [**View Results**](javascript:__doPostBack('ctl00$ctl00$FindField$FindField$historyControl$HistoryRepeater$ctl10$linkResults','')) (28,212)  [**View Details**](javascript:showShDetails(%22ctl00_ctl00_FindField_FindField_historyControl_ctrlPopup%22,%20%22S17%22);)  [**Edit**](http://web.a.ebscohost.com.ezproxy1.library.usyd.edu.au/Legacy/Views/UserControls/EHOST/) |
| S16 | (MH "Life Style, Sedentary+") | **Expanders** - Apply equivalent subjects  **Search modes** - Boolean/Phrase | [**View Results**](javascript:__doPostBack('ctl00$ctl00$FindField$FindField$historyControl$HistoryRepeater$ctl11$linkResults','')) (7,338)  [**View Details**](javascript:showShDetails(%22ctl00_ctl00_FindField_FindField_historyControl_ctrlPopup%22,%20%22S16%22);)  [**Edit**](http://web.a.ebscohost.com.ezproxy1.library.usyd.edu.au/Legacy/Views/UserControls/EHOST/) |
| S15 | (MH "Physical Activity") OR (MH "Exercise+") | **Expanders** - Apply equivalent subjects  **Search modes** - Boolean/Phrase | [**View Results**](javascript:__doPostBack('ctl00$ctl00$FindField$FindField$historyControl$HistoryRepeater$ctl12$linkResults','')) (130,542)  [**View Details**](javascript:showShDetails(%22ctl00_ctl00_FindField_FindField_historyControl_ctrlPopup%22,%20%22S15%22);)  [**Edit**](http://web.a.ebscohost.com.ezproxy1.library.usyd.edu.au/Legacy/Views/UserControls/EHOST/) |
| S14 | (MH "Pediatric Obesity") OR (MH "Obesity+") | **Expanders** - Apply equivalent subjects  **Search modes** - Boolean/Phrase | [**View Results**](javascript:__doPostBack('ctl00$ctl00$FindField$FindField$historyControl$HistoryRepeater$ctl13$linkResults','')) (89,778)  [**View Details**](javascript:showShDetails(%22ctl00_ctl00_FindField_FindField_historyControl_ctrlPopup%22,%20%22S14%22);)  [**Edit**](http://web.a.ebscohost.com.ezproxy1.library.usyd.edu.au/Legacy/Views/UserControls/EHOST/) |
| S13 | (MH "Nutrition+") | **Expanders** - Apply equivalent subjects  **Search modes** - Boolean/Phrase | [**View Results**](javascript:__doPostBack('ctl00$ctl00$FindField$FindField$historyControl$HistoryRepeater$ctl14$linkResults','')) (140,809)  [**View Details**](javascript:showShDetails(%22ctl00_ctl00_FindField_FindField_historyControl_ctrlPopup%22,%20%22S13%22);)  [**Edit**](http://web.a.ebscohost.com.ezproxy1.library.usyd.edu.au/Legacy/Views/UserControls/EHOST/) |
| S12 | (MH "Fast Foods") OR (MH "Snacks") OR (MH "Food+") | **Expanders** - Apply equivalent subjects  **Search modes** - Boolean/Phrase | [**View Results**](javascript:__doPostBack('ctl00$ctl00$FindField$FindField$historyControl$HistoryRepeater$ctl15$linkResults','')) (151,407)  [**View Details**](javascript:showShDetails(%22ctl00_ctl00_FindField_FindField_historyControl_ctrlPopup%22,%20%22S12%22);)  [**Edit**](http://web.a.ebscohost.com.ezproxy1.library.usyd.edu.au/Legacy/Views/UserControls/EHOST/) |
| S11 | (MH "Diet+") OR (MH "Diet, Western") | **Expanders** - Apply equivalent subjects  **Search modes** - Boolean/Phrase | [**View Results**](javascript:__doPostBack('ctl00$ctl00$FindField$FindField$historyControl$HistoryRepeater$ctl16$linkResults','')) (105,437)  [**View Details**](javascript:showShDetails(%22ctl00_ctl00_FindField_FindField_historyControl_ctrlPopup%22,%20%22S11%22);)  [**Edit**](http://web.a.ebscohost.com.ezproxy1.library.usyd.edu.au/Legacy/Views/UserControls/EHOST/) |
| S10 | S4 OR S5 OR S6 OR S7 OR S8 | **Expanders** - Apply equivalent subjects  **Search modes** - Boolean/Phrase | [**View Results**](javascript:__doPostBack('ctl00$ctl00$FindField$FindField$historyControl$HistoryRepeater$ctl17$linkResults','')) (159,608)  [**View Details**](javascript:showShDetails(%22ctl00_ctl00_FindField_FindField_historyControl_ctrlPopup%22,%20%22S10%22);)  [**Edit**](http://web.a.ebscohost.com.ezproxy1.library.usyd.edu.au/Legacy/Views/UserControls/EHOST/) |
| S9 | S1 OR S2 OR S3 | **Expanders** - Apply equivalent subjects  **Search modes** - Boolean/Phrase | [**View Results**](javascript:__doPostBack('ctl00$ctl00$FindField$FindField$historyControl$HistoryRepeater$ctl18$linkResults','')) (151,084)  [**View Details**](javascript:showShDetails(%22ctl00_ctl00_FindField_FindField_historyControl_ctrlPopup%22,%20%22S9%22);)  [**Edit**](http://web.a.ebscohost.com.ezproxy1.library.usyd.edu.au/Legacy/Views/UserControls/EHOST/) |
| S8 | (MH "Product Labeling+") OR (MH "Food Labeling") | **Expanders** - Also search within the full text of the articles; Apply equivalent subjects  **Search modes** - Boolean/Phrase | [**View Results**](javascript:__doPostBack('ctl00$ctl00$FindField$FindField$historyControl$HistoryRepeater$ctl19$linkResults','')) (11,976)  [**View Details**](javascript:showShDetails(%22ctl00_ctl00_FindField_FindField_historyControl_ctrlPopup%22,%20%22S8%22);)  [**Edit**](http://web.a.ebscohost.com.ezproxy1.library.usyd.edu.au/Legacy/Views/UserControls/EHOST/) |
| S7 | (MH "Taxes+") | **Expanders** - Also search within the full text of the articles; Apply equivalent subjects  **Search modes** - Boolean/Phrase | [**View Results**](javascript:__doPostBack('ctl00$ctl00$FindField$FindField$historyControl$HistoryRepeater$ctl20$linkResults','')) (6,570)  [**View Details**](javascript:showShDetails(%22ctl00_ctl00_FindField_FindField_historyControl_ctrlPopup%22,%20%22S7%22);)  [**Edit**](http://web.a.ebscohost.com.ezproxy1.library.usyd.edu.au/Legacy/Views/UserControls/EHOST/) |
| S6 | (MH "Paternalism") OR (MH "Social Norms") | **Expanders** - Also search within the full text of the articles; Apply equivalent subjects  **Search modes** - Boolean/Phrase | [**View Results**](javascript:__doPostBack('ctl00$ctl00$FindField$FindField$historyControl$HistoryRepeater$ctl21$linkResults','')) (2,474)  [**View Details**](javascript:showShDetails(%22ctl00_ctl00_FindField_FindField_historyControl_ctrlPopup%22,%20%22S6%22);)  [**Edit**](http://web.a.ebscohost.com.ezproxy1.library.usyd.edu.au/Legacy/Views/UserControls/EHOST/) |
| S5 | (MH "Government Regulations+") OR (MH "Rules and Regulations") | **Expanders** - Also search within the full text of the articles; Apply equivalent subjects  **Search modes** - Boolean/Phrase | [**View Results**](javascript:__doPostBack('ctl00$ctl00$FindField$FindField$historyControl$HistoryRepeater$ctl22$linkResults','')) (20,268)  [**View Details**](javascript:showShDetails(%22ctl00_ctl00_FindField_FindField_historyControl_ctrlPopup%22,%20%22S5%22);)  [**Edit**](http://web.a.ebscohost.com.ezproxy1.library.usyd.edu.au/Legacy/Views/UserControls/EHOST/) |
| S4 | (MH "Nutrition Policy+") OR (MH "Public Policy+") OR (MH "Policy Making") OR (MH "Health Policy+") OR (MH "Organizational Policies+") | **Expanders** - Also search within the full text of the articles; Apply equivalent subjects  **Search modes** - Boolean/Phrase | [**View Results**](javascript:__doPostBack('ctl00$ctl00$FindField$FindField$historyControl$HistoryRepeater$ctl23$linkResults','')) (124,870)  [**View Details**](javascript:showShDetails(%22ctl00_ctl00_FindField_FindField_historyControl_ctrlPopup%22,%20%22S4%22);)  [**Edit**](http://web.a.ebscohost.com.ezproxy1.library.usyd.edu.au/Legacy/Views/UserControls/EHOST/) |
| S3 | (MH "Health Beliefs") | **Expanders** - Also search within the full text of the articles; Apply equivalent subjects  **Search modes** - Boolean/Phrase | [**View Results**](javascript:__doPostBack('ctl00$ctl00$FindField$FindField$historyControl$HistoryRepeater$ctl24$linkResults','')) (12,349)  [**View Details**](javascript:showShDetails(%22ctl00_ctl00_FindField_FindField_historyControl_ctrlPopup%22,%20%22S3%22);)  [**Edit**](http://web.a.ebscohost.com.ezproxy1.library.usyd.edu.au/Legacy/Views/UserControls/EHOST/) |
| S2 | (MH "Attitude to Health+") OR (MH "Attitude to Obesity") | **Expanders** - Also search within the full text of the articles; Apply equivalent subjects  **Search modes** - Boolean/Phrase | [**View Results**](javascript:__doPostBack('ctl00$ctl00$FindField$FindField$historyControl$HistoryRepeater$ctl25$linkResults','')) (144,652)  [**View Details**](javascript:showShDetails(%22ctl00_ctl00_FindField_FindField_historyControl_ctrlPopup%22,%20%22S2%22);)  [**Edit**](http://web.a.ebscohost.com.ezproxy1.library.usyd.edu.au/Legacy/Views/UserControls/EHOST/) |
| S1 | (MH "Public Opinion") | **Expanders** - Also search within the full text of the articles; Apply equivalent subjects  **Search modes** - Boolean/Phrase | **View Results** (7,243)  [**View Details**](javascript:showShDetails(%22ctl00_ctl00_FindField_FindField_historyControl_ctrlPopup%22,%20%22S1%22);)  [**Edit**](http://web.a.ebscohost.com.ezproxy1.library.usyd.edu.au/Legacy/Views/UserControls/EHOST/) |
