# Supplementary material for: Measuring public opinion and acceptability of prevention policies: an integrative review and narrative synthesis of methods
Source: Health Res Policy Syst. 2022 Mar 4;20:26. doi: 10.1186/s12961-022-00829-y (PMC8895540; doi:10.1186/s12961-022-00829-y)
Supplement: Supplementary file 3 — Additional file 3: Studies included in review. [file 12961_2022_829_MOESM3_ESM.docx]

# Supplementary materials S3

## Studies included in review

| **Primary author** | **Year** | **Title of article** | **Country of study** | **Country region** | **Research type** | **Study design or method** | **Study design or method – details** | **Risk factor(s) or topic** | **Study population** | **Study population – details** | **Setting** |
| --- | --- | --- | --- | --- | --- | --- | --- | --- | --- | --- | --- |
| Aarts | 2011 | Feasibility of multi-sector policy measures that create activity-friendly environments for children: results of a Delphi study | The Netherlands | Europe | Mixed methods | Delphi study | Delphi study | Physical inactivity | Policy actors, influencers or stakeholders | Policy actors (eg. government officials) | Local |
| Agaku | 2014 | Support for smoke-free cars when children are present: a secondary analysis of 164,819 U.S. adults in 2010/2011 | United States | North America | Quantitative | Cross-sectional study - representative | Repeat or serial survey - single wave | Tobacco use and smoking | General public | General public | National |
| Agaku | 2015 | Effectiveness of text versus pictorial health warning labels and predictors of support for plain packaging of tobacco products within the European Union | Multiple countries | Multiple countries | Quantitative | Cross-sectional study - representative | Repeat or serial survey - single wave | Tobacco use and smoking | General public | General public | International |
| Agaku | 2019 | Adults' favorability toward prohibiting flavors in all tobacco products in the United States | United States | North America | Quantitative | Cross-sectional study - representative | Repeat or serial survey - single wave | Tobacco use and smoking | General public | General public | National |
| Ali | 2019 | U.S. Adults Attitudes Toward Lowering Nicotine Levels in Cigarettes | United States | North America | Quantitative | Cross-sectional study - representative | Repeat or serial survey - single wave | Tobacco use and smoking | General public | General public | National |
| Allman-Farinelli | 2019 | The Role of Supportive Food Environments to Enable Healthier Choices When Eating Meals Prepared Outside the Home: Findings from Focus Groups of 18 to 30-Year-Olds | Australia | Australia and New Zealand | Qualitative | Focus groups | Focus groups | Improve diet | Children, adolescents or young adults | Young adults | Local |
| Anaf | 2018 | A citizens jury on regulation of McDonald's products and operations in Australia in response to a corporate health impact assessment | Australia | Australia and New Zealand | Qualitative | Deliberative | Citizens' jury | Improve diet | General public | General public | Local |
| Arcand | 2013 | Results of a national survey examining Canadians' concern, actions, barriers, and support for dietary sodium reduction interventions | Canada | North America | Quantitative | Cross-sectional study - representative | Single survey | Improve diet | General public | General public | National |
| Astill Wright | 2019 | Understanding public opinion to the introduction of minimum unit pricing in Scotland: A qualitative study using Twitter | United Kingdom | UK and Ireland | Qualitative | Media analysis | Social media analysis | Alcohol use | Twitter users | Twitter users | State/regional |
| Avery | 2016 | Mechanisms of influence: Alcohol industry submissions to the inquiry into fetal alcohol spectrum disorders | Australia | Australia and New Zealand | Qualitative | Document or submission analysis | Submission analysis | Alcohol use | Policy actors, influencers or stakeholders | Retailers or industry | National |
| Bélanger-Gravel | 2015 | Implementing a public bicycle share program: Impact on perceptions and support for public policies for active transportation | Canada | North America | Quantitative | Cross-sectional study - representative | Repeat or serial survey - multiple waves | Physical inactivity | General public | General public | Local |
| Bélanger-Gravel | 2019 | Pattern and correlates of public support for public health interventions to reduce the consumption of sugar-sweetened beverages | Canada | North America | Quantitative | Cross-sectional study - representative | Single survey | Sugar-sweetened beverages | General public | General public | State/regional |
| Ball | 2017 | New Zealand policy experts' appraisal of interventions to reduce smoking in young adults: a qualitative investigation | New Zealand | Australia and New Zealand | Qualitative | Interviews | Interviews | Tobacco use and smoking | Policy actors, influencers or stakeholders | Policy actors, politicians, advocates & academics | National |
| Ballor | 2013 | Support for no-smoking policies among residents of public multiunit housing differs by smoking status | United States | North America | Quantitative | Cross-sectional study - representative | Single survey | Tobacco use and smoking | Other community group | Public housing residents | Local |
| Barry | 2011 | News media framing of childhood obesity in the United States from 2000 to 2009 | United States | North America | Qualitative | Media analysis | Media analysis | Overweight and obesity | News media | News media | National |
| Barry | 2013 | Taxes on sugar-sweetened beverages: Results from a 2011 national public opinion survey | United States | North America | Quantitative | Cross-sectional study - representative | Single survey | Sugar-sweetened beverages | General public | General public | National |
| Bates | 2018 | Awareness of alcohol as a risk factor for cancer is associated with public support for alcohol policies | United Kingdom | UK and Ireland | Quantitative | Cross-sectional study - representative | Single survey | Alcohol use | General public | General public | National |
| Beeken | 2013 | Public beliefs about the causes of obesity and attitudes towards policy initiatives in Great Britain | United Kingdom | UK and Ireland | Quantitative | Cross-sectional study - representative | Single survey | Overweight and obesity | General public | General public | National |
| Benowitz-Fredericks | 2018 | Voluntary Smoke-Free Measures Among Oklahoma Nightlife Owners: Barriers and Facilitators | United States | North America | Qualitative | Interviews | Interviews | Tobacco use and smoking | Policy actors, influencers or stakeholders | Retailers or industry | State/regional |
| Berg | 2011 | College student reactions to smoking bans in public, on campus and at home | United States | North America | Mixed methods | Multiple designs or methods | Cross-sectional survey; focus groups | Tobacco use and smoking | University/college students or staff | University/college students or staff | Local |
| Berg | 2016 | The Relationship between Weight-Based Prejudice and Attitudes towards Obesity-Reducing Public Policies | United States | North America | Quantitative | Cross-sectional study - convenience or purposive | Multiple surveys | Overweight and obesity | General public | General public | National |
| Berry | 2017 | Public attitudes to government intervention to regulate food advertising, especially to children | Australia | Australia and New Zealand | Quantitative | Cross-sectional study - representative | Repeat or serial survey - multiple waves | Improve diet | General public | General public | State/regional |
| Bhawra | 2018 | Are young Canadians supportive of proposed nutrition policies and regulations? An overview of policy support and the impact of socio-demographic factors on public opinion | Canada | North America | Quantitative | Cross-sectional study - convenience or purposive | Single survey | Improve diet | Children, adolescents or young adults | Young adults | National |
| Blumenthal | 2014 | Strategies to improve the dietary quality of Supplemental Nutrition Assistance Program (SNAP) beneficiaries: an assessment of stakeholder opinions | United States | North America | Quantitative | Cross-sectional study - convenience or purposive | Single survey | Improve diet | Policy actors, influencers or stakeholders | Stakeholders from academia, advocacy groups, government, health care and the food industry. | National |
| Boeckmann | 2018 | German public support for tobacco control policy measures: Results from the German study on tobacco use (DEBRA), a representative national survey | Germany | Europe | Quantitative | Cross-sectional study - representative | Repeat or serial survey - single wave | Tobacco use and smoking | General public | General public | National |
| Bolcic-Jankovic | 2015 | Public opinion about FDA regulation of menthol and nicotine | United States | North America | Quantitative | Cross-sectional study - representative | Single survey | Tobacco use and smoking | General public | General public | State/regional |
| Bonevski | 2011 | Support for smoke-free vocational education settings: an exploratory survey of staff behaviours, experiences and attitudes | Australia | Australia and New Zealand | Quantitative | Cross-sectional study - convenience or purposive | Single survey | Tobacco use and smoking | University/college students or staff | University/college students or staff | Local |
| Booth | 2019 | Headteachers' and chairs of governors' perspectives on adolescent obesity and its prevention in English secondary school settings | United Kingdom | UK and Ireland | Mixed methods | Multiple designs or methods | Cross-sectional survey; interviews | Overweight and obesity | Policy actors, influencers or stakeholders | Policy actors (eg. government officials) | State/regional |
| Bos | 2015 | Consumer Acceptance of Population-Level Intervention Strategies for Healthy Food Choices: The Role of Perceived Effectiveness and Perceived Fairness | The Netherlands | Europe | Quantitative | Cross-sectional study - representative | Single survey | Improve diet | General public | General public | National |
| Braverman | 2015 | Predictors of support among students, faculty and staff for a smoke-free university campus | United States | North America | Quantitative | Cross-sectional study - convenience or purposive | Single survey | Tobacco use and smoking | University/college students or staff | University/college students or staff | Local |
| Brennan | 2012 | Concept mapping: Priority community strategies to create changes to support active living | United States | North America | Mixed methods | Community-based participatory | Community-based participatory | Physical inactivity | Policy actors, influencers or stakeholders | Stakeholders including government, community and advocacy | Local |
| Brenner | 2018 | Attitudes towards smokefree high streets: a survey of local shoppers in a northern UK town | United Kingdom | UK and Ireland | Quantitative | Cross-sectional study - convenience or purposive | Single survey | Tobacco use and smoking | General public | General public | Local |
| Brown | 2012 | Adolescents' perceptions of tobacco control measures in the United Kingdom | United States | North America | Qualitative | Focus groups | Focus groups | Tobacco use and smoking | Children, adolescents or young adults | Children or adolescents | Local |
| Brown | 2012 | Support for removal of point-of-purchase tobacco advertising and displays: Findings from the International Tobacco Control (ITC) Canada survey | Canada | North America | Quantitative | Cohort study | Cohort study | Tobacco use and smoking | Smokers or former smokers | Smokers or former smokers | National |
| Buckton | 2018 | The palatability of sugar-sweetened beverage taxation: A content analysis of newspaper coverage of the UK sugar debate | United Kingdom | UK and Ireland | Mixed methods | Media analysis | Media analysis | Sugar-sweetened beverages | News media | News media | National |
| Burns | 2013 | 'Moving forward: a cross sectional baseline study of staff and student attitudes towards a totally smoke free university campus' | Australia | Australia and New Zealand | Quantitative | Cross-sectional study - convenience or purposive | Single survey | Tobacco use and smoking | University/college students or staff | University/college students or staff | Local |
| Burns | 2014 | An exploratory study of smokers' and stakeholders' expectations of the implementation of a smoke-free policy in a university setting | Australia | Australia and New Zealand | Qualitative | Interviews | Interviews | Tobacco use and smoking | University/college students or staff | University/college students or staff | Local |
| Buykx | 2015 | Public support for alcohol policies associated with knowledge of cancer risk | Australia | Australia and New Zealand | Quantitative | Cross-sectional study - representative | Single survey | Alcohol use | General public | General public | State/regional |
| Callinan | 2014 | Changes in Australian attitudes to alcohol policy: 1995-2010 | Australia | Australia and New Zealand | Quantitative | Cross-sectional study - representative | Repeat or serial survey - multiple waves | Alcohol use | General public | General public | National |
| Carlson | 2011 | Public support for street-scale urban design practices and policies to increase physical activity | United States | North America | Quantitative | Cross-sectional study - representative | Repeat or serial survey - single wave | Physical inactivity | General public | General public | National |
| Chambers | 2011 | What the UK public believe causes obesity, and what they want to do about it: A cross-sectional study | United Kingdom | UK and Ireland | Quantitative | Cross-sectional study - representative | Single survey | Overweight and obesity | General public | General public | National |
| Choi | 2013 | Young adults' support for adult-ratings for movies depicting smoking and for restrictions on tobacco magazine advertising | United States | North America | Quantitative | Cohort study | Cross-sectional substudy | Tobacco use and smoking | Children, adolescents or young adults | Children or adolescents | State/regional |
| Christensen | 2019 | Can a mass media campaign raise awareness of alcohol as a risk factor for cancer and public support for alcohol related policies? | Denmark | Europe | Quantitative | Cross-sectional study - representative | Repeat or serial survey - multiple waves | Alcohol use | General public | General public | National |
| Chung | 2012 | An analysis of potential barriers and enablers to regulating the television marketing of unhealthy foods to children at the state government level in Australia | Australia | Australia and New Zealand | Qualitative | Interviews | Interviews | Overweight and obesity | Policy actors, influencers or stakeholders | Policy actors (eg. government officials) | National |
| Claycomb | 2013 | Clean indoor air: Public demand for smoking bans | United States | North America | Quantitative | Cross-sectional study - convenience or purposive | Single survey | Tobacco use and smoking | General public | General public | Local |
| Cleland | 2013 | Obesity prevention programs and policies: practitioner and policy-maker perceptions of feasibility and effectiveness | Australia | Australia and New Zealand | Mixed methods | Cross-sectional study - convenience or purposive | Single survey | Overweight and obesity | Multiple groups | Government, NGO, medical practices, local community | State/regional |
| Cohn | 2015 | Reconceptualising public acceptability: A study of the ways people respond to policies aimed to reduce alcohol consumption | United Kingdom | UK and Ireland | Qualitative | Focus groups | Focus groups | Alcohol use | General public | General public | Local |
| Cole | 2019 | UK consumer perceptions of a novel till-receipt 'traffic-light' nutrition system | United Kingdom | UK and Ireland | Mixed methods | Cross-sectional study - convenience or purposive | Single survey | Improve diet | General public | General public | Local |
| Comans | 2017 | Public preferences for the use of taxation and labelling policy measures to combat obesity in young children in Australia | Australia | Australia and New Zealand | Quantitative | Cohort study | Cross-sectional substudy | Overweight and obesity | Parents or caregivers | Parents and caregivers | Local |
| Connolly | 2012 | Public attitudes regarding banning of cigarettes and regulation of nicotine | United States | North America | Quantitative | Cross-sectional study - representative | Single survey | Tobacco use and smoking | General public | General public | National |
| Coomber | 2017 | Predictors of awareness of standard drink labelling and drinking guidelines to reduce negative health effects among Australian drinkers | Australia | Australia and New Zealand | Quantitative | Cross-sectional study - representative | Single survey | Alcohol use | General public | General public | National |
| Cradock | 2018 | Driven to Support: Individual- and County-Level Factors Associated With Public Support for Active Transportation Policies | United States | North America | Quantitative | Cross-sectional study - representative | Single survey | Physical inactivity | General public | General public | Local |
| Curry | 2018 | Public Attitudes and Support for a Sugar-Sweetened Beverage Tax in America’s Heartland | United States | North America | Quantitative | Cross-sectional study - representative | Single survey | Sugar-sweetened beverages | General public | General public | State/regional |
| Czaplicki | 2019 | Support for E-Cigarette and Tobacco Control Policies Among Parents of Adolescents | United States | North America | Quantitative | Cross-sectional study - representative | Single survey | Tobacco use and smoking | Parents or caregivers | Parents or caregivers | National |
| D'Addezio | 2014 | Out-of-home eating frequency, causal attribution of obesity and support to healthy eating policies from a cross-European survey | Multiple countries | Multiple countries | Quantitative | Cross-sectional study - representative | Repeat or serial survey - single wave | Improve diet | General public | General public | International |
| Díez-Izquierdo | 2017 | Smoke-free homes and attitudes towards banning smoking in vehicles carrying children in Spain (2016) | Spain | Europe | Quantitative | Cross-sectional study - representative | Single survey | Tobacco use and smoking | General public | General public | National |
| Davó-Blanes | 2013 | The impact of marketing practices and its regulation policies on childhood obesity. Opinions of stakeholders in Spain | Spain | Europe | Qualitative | Interviews | Interviews | Overweight and obesity | Policy actors, influencers or stakeholders | public/government, private, media and NGO sectors | National |
| Davoren | 2019 | Support for evidence-based alcohol policy in Ireland: results from the Community Action on Alcohol Pilot Project | Ireland | UK and Ireland | Quantitative | Cross-sectional study - representative | Single survey | Alcohol use | General public | General public | Local |
| de Visser | 2014 | Which alcohol control strategies do young people think are effective? | United Kingdom | UK and Ireland | Quantitative | Cross-sectional study - convenience or purposive | Single survey | Alcohol use | Children, adolescents or young adults | Young adults | Local |
| Dinour | 2017 | Potato Chips, Cookies, and Candy Oh My! Public Commentary on Proposed Rules Regulating Competitive Foods | United States | North America | Mixed methods | Document or submission analysis | Public comment analysis | Improve diet | General public | General public | National |
| Donaldson | 2015 | Public support for a sugar-sweetened beverage tax and pro-tax messages in a Mid-Atlantic US state | United States | North America | Quantitative | Cross-sectional study - representative | Single survey | Sugar-sweetened beverages | General public | General public | State/regional |
| Dresler | 2013 | Attitudes of women from five European countries regarding tobacco control policies | Multiple countries | Multiple countries | Quantitative | Cross-sectional study - representative | Single survey | Tobacco use and smoking | General public | General public | International |
| Duffy | 2013 | Employee attitudes about moving toward a smoke-free campus at a Veterans Affairs hospital | United States | North America | Mixed methods | Cross-sectional study - convenience or purposive | Single survey | Tobacco use and smoking | Employees or managers | Employees | Local |
| Durant | 2018 | Changes in Sugary Beverage Consumption and Public Perceptions in Upstate New York After Implementation of a Community Awareness Campaign and Healthier Vending Strategies | United States | North America | Mixed methods | Multiple designs or methods | Cross-sectional survey; interviews | Sugar-sweetened beverages | General public | General public | Local |
| Edwards | 2012 | Qualitative exploration of public and smoker understanding of, and reactions to, an endgame solution to the tobacco epidemic | New Zealand | Australia and New Zealand | Qualitative | Focus groups | Focus groups | Tobacco use and smoking | General public | General public | Local |
| Edwards | 2013 | Support for a tobacco endgame and increased regulation of the tobacco industry among New Zealand smokers: results from a National Survey | New Zealand | Australia and New Zealand | Quantitative | Cohort study | Cross-sectional substudy | Tobacco use and smoking | Smokers or former smokers | Smokers or former smokers | National |
| Elliott-Green | 2016 | Sugar-sweetened beverages coverage in the British media: An analysis of public health advocacy versus pro-industry messaging | United Kingdom | UK and Ireland | Mixed methods | Media analysis | Media analysis | Sugar-sweetened beverages | News media | News media | National |
| Fabian | 2011 | Smoke-free laws in bars and restaurants: Does support among teens and young adults change after a statewide smoke-free law? | United States | North America | Quantitative | Cohort study | Cohort study | Tobacco use and smoking | Children, adolescents or young adults | Young adults | State/regional |
| Fairlie | 2015 | Local support for alcohol control policies and perceptions of neighborhood issues in two college communities | United States | North America | Quantitative | Cross-sectional study - convenience or purposive | Single survey | Alcohol use | University/college students or staff | University/college students or staff | Local |
| Farley | 2013 | Public opinions on tax and retail-based tobacco control strategies | United States | North America | Quantitative | Cross-sectional study - representative | Repeat or serial survey - multiple waves | Tobacco use and smoking | General public | General public | Local |
| Farley | 2014 | Public support for smoke-free air strategies among smokers and nonsmokers, New York City, 2010-2012 | United States | North America | Quantitative | Cross-sectional study - representative | Repeat or serial survey - multiple waves | Tobacco use and smoking | General public | General public | Local |
| Farrell | 2016 | Socio-economic divergence in public opinions about preventive obesity regulations: Is the purpose to 'make some things cheaper, more affordable' or to 'help them get over their own ignorance'? | Australia | Australia and New Zealand | Qualitative | Focus groups | Focus groups | Overweight and obesity | General public | General public | Local |
| Farrell | 2016 | Emotion in obesity discourse: Understanding public attitudes towards regulations for obesity prevention | Australia | Australia and New Zealand | Qualitative | Media analysis | Reader comment analysis | Overweight and obesity | News media | Reader commentary | National |
| Farrell | 2019 | Why do the public support or oppose obesity prevention regulations? Results from a South Australian population survey | Australia | Australia and New Zealand | Quantitative | Cross-sectional study - representative | Repeat or serial survey - single wave | Overweight and obesity | General public | General public | State/regional |
| Feng | 2017 | Twitter analysis of California’s failed campaign to raise the state’s tobacco tax by popular vote in 2012 | United States | North America | Qualitative | Media analysis | Social media analysis | Tobacco use and smoking | Twitter users | Twitter users | State/regional |
| Ferketich | 2016 | Relation between national-level tobacco control policies and individual-level voluntary home smoking bans in Europe | Multiple countries | Multiple countries | Quantitative | Cross-sectional study - representative | Repeat or serial survey - single wave | Tobacco use and smoking | General public | General public | International |
| Filippidis | 2017 | Are political views related to smoking and support for tobacco control policies? A survey across 28 European countries | Multiple countries | Multiple countries | Quantitative | Cross-sectional study - representative | Repeat or serial survey - single wave | Tobacco use and smoking | General public | General public | International |
| Fix | 2011 | Smokers' reactions to FDA regulation of tobacco products: findings from the 2009 ITC United States survey | United States | North America | Quantitative | Cohort study | Cross-sectional substudy | Tobacco use and smoking | Smokers or former smokers | Smokers or former smokers | National |
| Fogarty | 2012 | Advocates, interest groups and Australian news coverage of alcohol advertising restrictions: Content and framing analysis | Australia | Australia and New Zealand | Mixed methods | Media analysis | Media analysis | Alcohol use | News media | News media | National |
| Fogarty | 2013 | "Like Throwing a Bowling Ball at a Battle Ship" Audience Responses to Australian News Stories about Alcohol Pricing and Promotion Policies: A Qualitative Focus Group Study | Australia | Australia and New Zealand | Qualitative | Focus groups | Focus groups | Alcohol use | General public | General public | Local |
| Fogarty | 2013 | What should be done about policy on alcohol pricing and promotions? Australian experts' views of policy priorities: a qualitative interview study | Australia | Australia and New Zealand | Qualitative | Interviews | Interviews | Alcohol use | Policy actors, influencers or stakeholders | Academics; policy and NGO advocates | National |
| Foltz | 2012 | Support among U.S. adults for local and state policies to increase fruit and vegetable access | United States | North America | Quantitative | Cross-sectional study - representative | Repeat or serial survey - single wave | Improve diet | General public | General public | National |
| Fong | 2013 | Evaluating the Effectiveness of France's Indoor Smoke-Free Law 1 Year and 5 Years after Implementation: Findings from the ITC France Survey | France | Europe | Quantitative | Cohort study | Cohort study | Tobacco use and smoking | Multiple groups | General public; smokers or former smokers | National |
| Ford | 2020 | Parents' and carers' awareness and perceptions of UK supermarket policies on less healthy food at checkouts: A qualitative study | United Kingdom | UK and Ireland | Qualitative | Focus groups | Focus groups | Improve diet | Parents or caregivers | Parents or caregivers | Local |
| Foster | 2012 | Agenda-building influences on the news media's coverage of the U.S. Food and Drug Administration's push to regulate tobacco, 1993-2009 | United States | North America | Mixed methods | Media analysis | Media analysis | Tobacco use and smoking | News media | News media | National |
| Fraser | 2017 | Perspectives of key stakeholders and smokers on a very low nicotine content cigarette-only policy: qualitative study | New Zealand | Australia and New Zealand | Qualitative | Multiple designs or methods | Interviews; focus groups | Tobacco use and smoking | Multiple groups | Stakeholders (politicians, government health officials, commercial/industry); smokers | Local |
| Freeman | 2011 | Tobacco plain packaging legislation: A content analysis of commentary posted on Australian online news | Australia | Australia and New Zealand | Mixed methods | Media analysis | Reader comment analysis | Tobacco use and smoking | News media | Reader commentary | National |
| Fu | 2018 | Correlates of the support for smoke-free policies among smokers: A cross-sectional study in six European countries of the EUREST-PLUS ITC EUROPE SURVEYS | Multiple countries | Multiple countries | Quantitative | Cohort study | Cross-sectional substudy | Tobacco use and smoking | Smokers or former smokers | Smokers or former smokers | International |
| Gallus | 2014 | Support for a tobacco endgame strategy in 18 European countries | Multiple countries | Multiple countries | Quantitative | Cross-sectional study - representative | Single survey | Tobacco use and smoking | General public | General public | International |
| Gardner | 2017 | How did the public respond to the 2015 expert consensus public health guidance statement on workplace sedentary behaviour? A qualitative analysis | United Kingdom | UK and Ireland | Qualitative | Media analysis | Reader comment analysis | Physical inactivity | News media | Reader commentary | National |
| Gase | 2015 | Public awareness of and support for infrastructure changes designed to increase walking and biking in Los Angeles County | United States | North America | Quantitative | Cross-sectional study - convenience or purposive | Single survey | Physical inactivity | General public | General public | Local |
| Gendall | 2013 | Public support for more action on smoking | New Zealand | Australia and New Zealand | Quantitative | Cross-sectional study - representative | Single survey | Tobacco use and smoking | General public | General public | National |
| Gendall | 2015 | Should support for obesity interventions or perceptions of their perceived effectiveness shape policy? | New Zealand | Australia and New Zealand | Quantitative | Cross-sectional study - representative | Single survey | Overweight and obesity | General public | General public | National |
| Gentzke | 2020 | Adults' attitudes toward raising the minimum age of sale for tobacco products to 21 years, United States, 2014-2017 | United States | North America | Quantitative | Cross-sectional study - representative | Repeat or serial survey - multiple waves | Tobacco use and smoking | General public | General public | National |
| Goldthorpe | 2019 | Who is responsible for keeping children healthy? A qualitative exploration of the views of children aged 8-10 years old | United Kingdom | UK and Ireland | Qualitative | Focus groups | Focus groups | Preventive health | Children, adolescents or young adults | Children or adolescents | Local |
| Gollust | 2014 | Americans' opinions about policies to reduce consumption of sugar-sweetened beverages | United States | North America | Quantitative | Cross-sectional study - representative | Single survey | Sugar-sweetened beverages | General public | General public | National |
| Greenfield | 2014 | Second-hand drinking may increase support for alcohol policies: New results from the 2010 National Alcohol Survey | United States | North America | Quantitative | Cross-sectional study - representative | Repeat or serial survey - single wave | Alcohol use | General public | General public | National |
| Grunseit | 2019 | Nanny or canny? Community perceptions of government intervention for preventive health | Australia | Australia and New Zealand | Mixed methods | Multiple designs or methods | Cross-sectional survey; focus groups | Preventive health | General public | General public | National |
| Gustat | 2019 | Perceptions of the built environment and support for policies that promote physical activity | United States | North America | Quantitative | Cross-sectional study - convenience or purposive | Single survey | Physical inactivity | General public | General public | State/regional |
| Hübner | 2012 | Swedish public opinion on alcohol and alcohol policy, 1995 and 2003 | Sweden | Europe | Quantitative | Cross-sectional study - representative | Repeat or serial survey - multiple waves | Alcohol use | General public | General public | National |
| Hale | 2017 | Effect of a smoke-free policy on staff attitudes and behaviours within an Australian metropolitan health service: a 3 year cross-sectional study | Australia | Australia and New Zealand | Quantitative | Cross-sectional study - convenience or purposive | Multiple surveys | Tobacco use and smoking | Employees or managers | Employees | Local |
| Hall | 2015 | Assessment of Attitudes Regarding Tobacco-Free Campus Policy and Secondhand Smoke Exposure Among College Students, Faculty, and Staff | United States | North America | Quantitative | Cross-sectional study - convenience or purposive | Single survey | Tobacco use and smoking | University/college students or staff | University/college students or staff | Local |
| Hayes | 2014 | Public opinion about ending the sale of tobacco in Australia | Australia | Australia and New Zealand | Quantitative | Cross-sectional study - representative | Repeat or serial survey - multiple waves | Tobacco use and smoking | General public | General public | State/regional |
| Haynes-Maslow | 2015 | Low-income individuals' perceptions about fruit and vegetable access programs: A qualitative study | United States | North America | Qualitative | Focus groups | Focus groups | Improve diet | General public | General public | Local |
| Henderson | 2013 | Evaluating the use of citizens' juries in food policy: A case study of food regulation | Australia | Australia and New Zealand | Qualitative | Deliberative | Citizens' jury | Improve diet | General public | General public | Local |
| Hewett | 2012 | Secondhand smoke and smokefree policies in owner-occupied multi-unit housing | United States | North America | Quantitative | Cross-sectional study - representative | Single survey | Tobacco use and smoking | Other community group | Multi unit housing residents | Local |
| Hildebrand | 2019 | Parents’ Perceptions of Childhood Obesity and Support of the School Wellness Policy | United States | North America | Quantitative | Cross-sectional study - convenience or purposive | Single survey | Overweight and obesity | Parents or caregivers | Parents or caregivers | Local |
| Hilton | 2012 | Escalating coverage of obesity in UK newspapers: The evolution and framing of the obesity epidemic from 1996 to 2010 | United Kingdom | UK and Ireland | Mixed methods | Media analysis | Media analysis | Overweight and obesity | News media | News media | National |
| Hilton | 2014 | Implications for alcohol minimum unit pricing advocacy: What can we learn for public health from UK newsprint coverage of key claim-makers in the policy debate? | United Kingdom | UK and Ireland | Mixed methods | Media analysis | Media analysis | Alcohol use | News media | News media | National |
| Hilton | 2014 | Newsprint coverage of smoking in cars carrying children: A case study of public and scientific opinion driving the policy debate | United Kingdom | UK and Ireland | Mixed methods | Media analysis | Media analysis | Tobacco use and smoking | News media | News media | National |
| Hilton | 2019 | Following in the footsteps of tobacco and alcohol? Stakeholder discourse in UK newspaper coverage of the Soft Drinks Industry Levy | United Kingdom | UK and Ireland | Mixed methods | Media analysis | Media analysis | Sugar-sweetened beverages | News media | News media | National |
| Hitchman | 2011 | Support and correlates of support for banning smoking in cars with children: Findings from the ITC Four Country Survey | Multiple countries | Multiple countries | Quantitative | Cohort study | Cross-sectional substudy | Tobacco use and smoking | Smokers or former smokers | Smokers or former smokers | International |
| Hoek | 2012 | Strong public support for plain packaging of tobacco products | New Zealand | Australia and New Zealand | Quantitative | Cross-sectional study - representative | Single survey | Tobacco use and smoking | General public | General public | National |
| Hoek | 2016 | A qualitative analysis of low income smokers’ responses to tobacco excise tax increases | New Zealand | Australia and New Zealand | Qualitative | Interviews | Interviews | Tobacco use and smoking | Smokers or former smokers | Smokers or former smokers | Local |
| Hood | 2013 | Individual, social, and environmental factors associated with support for smoke-free housing policies among subsidized multiunit housing tenants | United States | North America | Quantitative | Cross-sectional study - convenience or purposive | Single survey | Tobacco use and smoking | General public | General public | Local |
| Hope | 2014 | The ebb and flow of attitudes and policies on alcohol in Ireland 2002-2010 | Ireland | UK and Ireland | Quantitative | Cross-sectional study - representative | Repeat or serial survey - multiple waves | Alcohol use | General public | General public | National |
| Howse | 2017 | The university should promote health, but not enforce it': Opinions and attitudes about the regulation of sugar-sweetened beverages in a university setting | Australia | Australia and New Zealand | Mixed methods | Cross-sectional study - convenience or purposive | Single survey | Sugar-sweetened beverages | University/college students or staff | University/college students or staff | Local |
| Huse | 2019 | Recreation centre managers' perceptions of pricing interventions to promote healthy eating | Australia | Australia and New Zealand | Qualitative | Interviews | Interviews | Improve diet | Policy actors, influencers or stakeholders | Recreation centre managers | State/regional |
| Ialomiteanu | 2014 | Trends in public opinion on alcohol issues during a period of increasing access to alcohol: Ontario, Canada, 1996-2011 | Canada | North America | Quantitative | Cross-sectional study - representative | Repeat or serial survey - multiple waves | Alcohol use | General public | General public | State/regional |
| Ickes | 2017 | Students' Beliefs About and Perceived Effectiveness of a Tobacco-Free Campus Policy | United States | North America | Quantitative | Cross-sectional study - convenience or purposive | Single survey | Tobacco use and smoking | University/college students or staff | University/college students or staff | Local |
| Ickes | 2019 | Support for Tobacco 21 in a Tobacco-Growing State | United States | North America | Quantitative | Cross-sectional study - representative | Single survey | Tobacco use and smoking | General public | General public | State/regional |
| Jürkenbeck | 2020 | Nutrition policy and individual struggle to eat healthily: The question of public support | Germany | Europe | Quantitative | Cross-sectional study - representative | Single survey | Improve diet | General public | General public | National |
| Jaine | 2014 | New Zealand tobacco retailers' attitudes to selling tobacco, point-of-sale display bans and other tobacco control measures: a qualitative analysis | New Zealand | Australia and New Zealand | Qualitative | Interviews | Interviews | Tobacco use and smoking | Policy actors, influencers or stakeholders | Retailers or industry | Local |
| Jaine | 2015 | How adolescents view the tobacco endgame and tobacco control measures: trends and associations in support among 14-15 year olds | New Zealand | Australia and New Zealand | Quantitative | Cross-sectional study - representative | Repeat or serial survey - multiple waves | Tobacco use and smoking | Children, adolescents or young adults | Children or adolescents | National |
| Jeong | 2014 | Local News Media Framing of Obesity in the Context of a Sugar-Sweetened Beverage Reduction Media Campaign | United States | North America | Mixed methods | Media analysis | Media analysis | Sugar-sweetened beverages | News media | News media | Local |
| Jones | 2012 | It's all in the lens: Differences in views on obesity prevention between advocates and policy makers | United States | North America | Qualitative | Interviews | Interviews | Overweight and obesity | Policy actors, influencers or stakeholders | Legislators/politicians/policymakers; advocates | State/regional |
| Julia | 2015 | Public perception and characteristics related to acceptance of the sugar-sweetened beverage taxation launched in France in 2012 | France | Europe | Quantitative | Cohort study | Cross-sectional substudy | Sugar-sweetened beverages | General public | General public | National |
| Kamyab | 2015 | Public support for graphic health warning labels in the U.S | United States | North America | Quantitative | Cross-sectional study - representative | Repeat or serial survey - multiple waves | Tobacco use and smoking | General public | General public | National |
| Kandra | 2013 | Support among middle school and high school students for smoke-free policies, North Carolina, 2009 | United States | North America | Quantitative | Cross-sectional study - convenience or purposive | Multiple surveys | Tobacco use and smoking | Children, adolescents or young adults | Children or adolescents | State/regional |
| Kang | 2017 | The public's opinions on a new school meals policy for childhood obesity prevention in the U.S.: A social media analytics approach | United States | North America | Mixed methods | Media analysis | Social media analysis | Overweight and obesity | Twitter users | Twitter users | National |
| Katikireddi | 2015 | How did policy actors use mass media to influence the Scottish alcohol minimum unit pricing debate? Comparative analysis of newspapers, evidence submissions and interviews | United Kingdom | UK and Ireland | Qualitative | Multiple designs or methods | Media analysis; document analysis; evidence submissions; interviews. | Alcohol use | Multiple groups | Media analysis; interviews with policy actors (academics, advocates, industry reps, politicians, civil servants) | National |
| Keatley | 2018 | Attitudes and beliefs towards alcohol minimum pricing in Western Australia | Australia | Australia and New Zealand | Qualitative | Focus groups | Focus groups | Alcohol use | General public | General public | State/regional |
| Kelly | 2012 | Restricting unhealthy food sponsorship: Attitudes of the sporting community | Australia | Australia and New Zealand | Quantitative | Cross-sectional study - convenience or purposive | Single survey | Improve diet | Multiple groups | Sports clubs officials; parents; sporting associations | State/regional |
| Kelly | 2013 | Views of children and parents on limiting unhealthy food, drink and alcohol sponsorship of elite and children's sports | Australia | Australia and New Zealand | Quantitative | Cross-sectional study - convenience or purposive | Single survey | Preventive health | Multiple groups | Parents; children | State/regional |
| Kilian | 2019 | How attitudes toward alcohol policies differ across European countries: Evidence from the standardized european alcohol survey (seas) | Multiple countries | Multiple countries | Quantitative | Cross-sectional study - representative | Repeat or serial survey - multiple waves | Alcohol use | General public | General public | International |
| Killian | 2020 | State Lawmaker's Views on Childhood Obesity and Related School Wellness Legislation | United States | North America | Qualitative | Interviews | Interviews | Overweight and obesity | Policy actors, influencers or stakeholders | Political representatives | State/regional |
| Kim | 2019 | Public support for health taxes and media regulation of harmful products in South Korea | South Korea | Asia | Quantitative | Cross-sectional study - representative | Single survey | Preventive health | General public | General public | National |
| King | 2013 | Attitudes toward smoke-free workplaces, restaurants, and bars, casinos, and clubs among U.S. adults: Findings from the 2009-2010 national adult tobacco survey | United States | North America | Quantitative | Cross-sectional study - representative | Repeat or serial survey - single wave | Tobacco use and smoking | General public | General public | National |
| Kirst | 2017 | Addressing health inequities in Ontario, Canada: what solutions do the public support? | Canada | North America | Quantitative | Cross-sectional study - representative | Single survey | Health equity | General public | General public | State/regional |
| Kongats | 2020 | Using the intervention ladder to examine policy influencer and general public support for potential tobacco control policies in Alberta and Quebec | Canada | North America | Quantitative | Cross-sectional study - representative | Repeat or serial survey - single wave | Tobacco use and smoking | Multiple groups | General public; Policy actors & influencers; politicians | State/regional |
| Kroon | 2013 | Public perceptions of the ban on tobacco sales in San Francisco pharmacies | United States | North America | Quantitative | Cross-sectional study - convenience or purposive | Single survey | Tobacco use and smoking | General public | General public | Local |
| Kruger | 2015 | National and state attitudes of US adults toward tobacco-free school grounds, 2009-2010 | United States | North America | Quantitative | Cross-sectional study - representative | Repeat or serial survey - single wave | Tobacco use and smoking | General public | General public | National |
| Kruger | 2016 | National and state-specific attitudes toward smoke-free parks among U.S. Adults | United States | North America | Quantitative | Cross-sectional study - representative | Repeat or serial survey - single wave | Tobacco use and smoking | General public | General public | National |
| Krukowski | 2016 | A qualitative study of adolescent views of sugar-sweetened beverage Taxes, Michigan, 2014 | United States | North America | Qualitative | Focus groups | Focus groups | Sugar-sweetened beverages | Children, adolescents or young adults | Children or adolescents | Local |
| Kuijpers | 2018 | Public support for tobacco control policies: The role of the protection of children against tobacco | The Netherlands | Europe | Quantitative | Cross-sectional study - representative | Single survey | Tobacco use and smoking | General public | General public | National |
| Kuiper | 2013 | Newspaper coverage of implementation of the Michigan smoke-free law: Lessons learned | United States | North America | Qualitative | Media analysis | Media analysis | Tobacco use and smoking | News media | News media | State/regional |
| Kwon | 2019 | A multi-country survey of public support for food policies to promote healthy diets: Findings from the International Food Policy Study | Multiple countries | Multiple countries | Quantitative | Cohort study | Cross-sectional substudy | Improve diet | General public | General public | International |
| Kypri | 2014 | Public, official, and industry submissions on a Bill to increase the alcohol minimum purchasing age: A critical analysis | New Zealand | Australia and New Zealand | Qualitative | Document or submission analysis | Submission analysis | Alcohol use | Multiple groups | General public; industry; NGOs. | National |
| Ladekjær Larsen | 2016 | Students' drinking behavior and perceptions towards introducing alcohol policies on university campus in Denmark: a focus group study | Denmark | Europe | Qualitative | Focus groups | Focus groups | Alcohol use | University/college students or staff | University/college students or staff | Local |
| Laverty | 2018 | Associations of e-cigarette experimentation with support for tobacco control policies in the European Union, 2012-2014 | Multiple countries | Multiple countries | Quantitative | Cross-sectional study - representative | Repeat or serial survey - multiple waves | Tobacco use and smoking | General public | General public | International |
| Lee | 2013 | Public Views on Food Addiction and Obesity: Implications for Policy and Treatment | Multiple countries | Multiple countries | Mixed methods | Cross-sectional study - convenience or purposive | Single survey | Overweight and obesity | General public | General public | International |
| Leung | 2019 | Perspectives from Supplemental Nutrition Assistance Program Participants on Improving SNAP Policy | United States | North America | Mixed methods | Cross-sectional study - convenience or purposive | Single survey | Improve diet | Other community group | SNAP participants; food insecure participants | National |
| Li | 2017 | Public attitudes towards alcohol control policies in Scotland and England: Results from a mixed-methods study | United Kingdom | UK and Ireland | Mixed methods | Multiple designs or methods | Cross-sectional survey; focus groups | Alcohol use | General public | General public | National |
| Licht | 2012 | Attitudes, Experiences, and Acceptance of Smoke-Free Policies Among US Multiunit Housing Residents | United States | North America | Quantitative | Cross-sectional study - representative | Single survey | Tobacco use and smoking | Other community group | Multi unit housing residents | National |
| Lidon-Moyano | 2018 | Attitudes towards tobacco product regulations and their relationship with the tobacco control policies | Multiple countries | Multiple countries | Quantitative | Cross-sectional study - representative | Repeat or serial survey - multiple waves | Tobacco use and smoking | General public | General public | International |
| Livingston | 2019 | The impact of high profile restrictions on support for alcohol control policies | Australia | Australia and New Zealand | Quantitative | Cross-sectional study - representative | Repeat or serial survey - multiple waves | Alcohol use | General public | General public | National |
| Long | 2014 | Public support for policies to improve the nutritional impact of the Supplemental Nutrition Assistance Program (SNAP) | United States | North America | Quantitative | Cross-sectional study - representative | Single survey | Improve diet | General public | General public | National |
| Lonsdale | 2012 | A minimum price per unit of alcohol: A focus group study to investigate public opinion concerning UK government proposals to introduce new price controls to curb alcohol consumption | United Kingdom | UK and Ireland | Qualitative | Focus groups | Focus groups | Alcohol use | General public | General public | State/regional |
| Luecking | 2017 | Impact of Weight of the Nation Community Screenings on Obesity-Related Beliefs | United States | North America | Quantitative | Cross-sectional study - convenience or purposive | Multiple surveys | Overweight and obesity | General public | General public | Local |
| Lund | 2016 | Exploring Smokers' Opposition to Proposed Tobacco Control Strategies | Norway | Europe | Quantitative | Cross-sectional study - representative | Single survey | Tobacco use and smoking | General public | General public | National |
| Lund | 2016 | Overlap in attitudes to policy measures on alcohol, tobacco and illegal drugs | Norway | Europe | Quantitative | Cross-sectional study - representative | Single survey | Preventive health | General public | General public | National |
| Lykke | 2014 | Temporal changes in the attitude towards smoking bans in public arenas among adults in the Capital Region of Denmark from 2007 to 2010 | Denmark | Europe | Quantitative | Cross-sectional study - representative | Repeat or serial survey - multiple waves | Tobacco use and smoking | General public | General public | State/regional |
| Lykke | 2016 | Ready for a goodbye to tobacco? - Assessment of support for endgame strategies on smoking among adults in a Danish regional health survey | Denmark | Europe | Quantitative | Cross-sectional study - representative | Single survey | Tobacco use and smoking | General public | General public | State/regional |
| Macdonald | 2011 | The relationship between alcohol problems, perceived risks and attitudes toward alcohol policy in Canada | Canada | North America | Quantitative | Cross-sectional study - representative | Repeat or serial survey - single wave | Alcohol use | General public | General public | National |
| Maclennan | 2012 | Non-response bias in a community survey of drinking, alcohol-related experiences and public opinion on alcohol policy | New Zealand | Australia and New Zealand | Quantitative | Cross-sectional study - representative | Single survey | Alcohol use | General public | General public | State/regional |
| Maclennan | 2012 | Public sentiment towards alcohol and local government alcohol policies in New Zealand | New Zealand | Australia and New Zealand | Quantitative | Cross-sectional study - convenience or purposive | Single survey | Alcohol use | General public | General public | State/regional |
| Macy | 2012 | Smoking behaviors and attitudes during adolescence prospectively predict support for tobacco control policies in adulthood | United States | North America | Quantitative | Cohort study | Cohort study | Tobacco use and smoking | General public | General public | Local |
| Macy | 2013 | The association between implicit and explicit attitudes toward smoking and support for tobacco control measures | United States | North America | Quantitative | Cohort study | Cross-sectional substudy | Tobacco use and smoking | General public | General public | Local |
| Mah | 2013 | Ready for policy? stakeholder attitudes toward menu labelling in Toronto, Canada | Canada | North America | Mixed methods | Multiple designs or methods | Survey; interviews; policy consultation | Improve diet | Multiple groups | General public; retailers - restaurant operators, chain and franchise operators, retailer association | Local |
| Mammen | 2012 | Understanding the drive to escort: A cross-sectional analysis examining parental attitudes towards children's school travel and independent mobility | Canada | North America | Quantitative | Cross-sectional study - representative | Single survey | Physical inactivity | General public | General public | Local |
| March-Cerdá | 2013 | Acceptability and Impact of Measures Regulating Alcohol Consumption Among Adolescents in Spain: Opinions of Adolescents, Parents, and Teachers | Spain | Europe | Mixed methods | Multiple designs or methods | Cross-sectional survey; focus groups; interviews | Alcohol use | Multiple groups | Adolescents; parents; teachers | National |
| Marsh | 2014 | Attitudes towards smokefree campus policies in New Zealand | New Zealand | Australia and New Zealand | Quantitative | Cross-sectional study - convenience or purposive | Single survey | Tobacco use and smoking | University/college students or staff | University/college students or staff | Local |
| Martínez-Sánchez | 2014 | Smoking while driving and public support for car smoking bans in Italy | Italy | Europe | Quantitative | Cross-sectional study - representative | Single survey | Tobacco use and smoking | General public | General public | National |
| Mata | 2018 | Public beliefs about obesity relative to other major health risks: Representative cross-sectional surveys in the USA, the UK, and Germany | Multiple countries | Multiple countries | Quantitative | Cross-sectional study - representative | Single survey | Preventive health | General public | General public | International |
| Mazzocchi | 2014 | What is the public appetite for healthy eating policies? Evidence from a cross-European survey | Multiple countries | Multiple countries | Quantitative | Cross-sectional study - representative | Single survey | Improve diet | General public | General public | International |
| McDaniel | 2015 | "Tired of watching customers walk out the door because of the smoke": A content analysis of media coverage of voluntarily smokefree restaurants and bars | United States | North America | Mixed methods | Media analysis | Media analysis | Tobacco use and smoking | News media | News media | National |
| McEvoy | 2014 | Adolescents' views about a proposed rewards intervention to promote healthy food choice in secondary school canteens | United Kingdom | UK and Ireland | Qualitative | Focus groups | Focus groups | Improve diet | Children, adolescents or young adults | Children or adolescents | Local |
| McGetrick | 2019 | Healthy public policy options to promote physical activity for chronic disease prevention: Understanding Canadian policy influencer and general public preferences | Canada | North America | Quantitative | Cross-sectional study - representative | Single survey | Physical inactivity | Multiple groups | General public; Policy actors; politicians. | State/regional |
| McMillen | 2018 | Adult attitudes and practices regarding smoking restrictions and child tobacco smoke exposure: 2000 to 2015 | United States | North America | Quantitative | Cross-sectional study - representative | Repeat or serial survey - multiple waves | Tobacco use and smoking | General public | General public | National |
| McMillen | 2019 | Public Support for Smoke-Free Section 8 Public Housing | United States | North America | Quantitative | Cross-sectional study - representative | Repeat or serial survey - single wave | Tobacco use and smoking | General public | General public | National |
| Miller | 2019 | Are Australians ready for warning labels, marketing bans and sugary drink taxes? Two cross-sectional surveys measuring support for policy responses to sugar-sweetened beverages | Australia | Australia and New Zealand | Quantitative | Cross-sectional study - representative | Single survey | Sugar-sweetened beverages | General public | General public | State/regional |
| Mons | 2012 | Comprehensive smoke-free policies attract more support from smokers in Europe than partial policies | Multiple countries | Multiple countries | Quantitative | Cohort study | Cohort study | Tobacco use and smoking | Smokers or former smokers | Smokers or former smokers | International |
| Moodie | 2017 | Adolescents' Perceptions of an On-cigarette Health Warning | United Kingdom | UK and Ireland | Quantitative | Cross-sectional study - representative | Repeat or serial survey - single wave | Tobacco use and smoking | Children, adolescents or young adults | Children or adolescents | National |
| Moore | 2012 | Support for tobacco control interventions: do country of origin and socioeconomic status make a difference? | Multiple countries | Multiple countries | Quantitative | Cohort study | Cross-sectional substudy | Tobacco use and smoking | Smokers or former smokers | Smokers or former smokers | International |
| Moore | 2019 | Determinants of support for government involvement in obesity control among American adults | United States | North America | Quantitative | Cross-sectional study - representative | Single survey | Overweight and obesity | General public | General public | National |
| Morain | 2013 | Survey finds public support for legal interventions directed at health behavior to fight noncommunicable disease | United States | North America | Quantitative | Cross-sectional study - representative | Single survey | Preventive health | General public | General public | National |
| Morain | 2018 | State-level support for tobacco 21 Laws: Results of a five-state survey | United States | North America | Quantitative | Cross-sectional study - representative | Single survey | Tobacco use and smoking | General public | General public | State/regional |
| Moran | 2016 | Believing that certain foods are addictive is associated with support for obesity-related public policies | United States | North America | Quantitative | Cross-sectional study - representative | Single survey | Preventive health | General public | General public | National |
| Moretto | 2014 | Yes, the government should tax soft drinks: Findings from a citizens' Jury in Australia | Australia | Australia and New Zealand | Qualitative | Deliberative | Citizens' jury | Sugar-sweetened beverages | General public | General public | Local |
| Morley | 2012 | Public opinion on food-related obesity prevention policy initiatives | Australia | Australia and New Zealand | Quantitative | Cross-sectional study - representative | Single survey | Overweight and obesity | General public | General public | National |
| Moshrefzadeh | 2013 | A content analysis of media coverage of the introduction of a smoke-free bylaw in Vancouver parks and beaches | Canada | North America | Mixed methods | Media analysis | Media analysis | Tobacco use and smoking | News media | News media | Local |
| Nagelhout | 2015 | Do smokers support smoke-free laws to help themselves quit smoking? Findings from a longitudinal study | United States | North America | Quantitative | Cross-sectional study - representative | Repeat or serial survey - multiple waves | Tobacco use and smoking | Smokers or former smokers | Smokers or former smokers | National |
| Nagelhout | 2015 | Population support before and after the implementation of smoke-free laws in the United States: Trends from 1992-2007 | United States | North America | Quantitative | Cross-sectional study - representative | Repeat or serial survey - multiple waves | Tobacco use and smoking | Smokers or former smokers | Smokers or former smokers | National |
| Niederdeppe | 2011 | Qualitative research about attributions, narratives, and support for obesity policy, 2008 | United States | North America | Qualitative | Focus groups | Focus groups | Overweight and obesity | General public | General public | Local |
| Niederdeppe | 2013 | News Coverage of Sugar-Sweetened Beverage Taxes: Pro- and Antitax Arguments in Public Discourse | United States | North America | Mixed methods | Media analysis | Media analysis | Sugar-sweetened beverages | News media | News media | State/regional |
| Nikitas | 2016 | The paradox of public acceptance of bike sharing in Gothenburg | Sweden | Europe | Mixed methods | Cross-sectional study - convenience or purposive | Single survey | Physical inactivity | General public | General public | Local |
| Nykiforuk | 2014 | Cancer beliefs and prevention policies: comparing Canadian decision-maker and general population views | Canada | North America | Quantitative | Cross-sectional study - convenience or purposive | Single survey | Preventive health | Multiple groups | General public; policy influencers. | State/regional |
| Oddo | 2019 | Perceptions of the possible health and economic impacts of Seattle's sugary beverage tax | United States | North America | Quantitative | Cross-sectional study - representative | Single survey | Sugar-sweetened beverages | General public | General public | Local |
| Okoli | 2013 | Support for a smoke-free bylaw in parks and on beaches | Canada | North America | Quantitative | Cross-sectional study - convenience or purposive | Single survey | Tobacco use and smoking | General public | General public | Local |
| Olsen | 2018 | Physical activity and sedentary behaviour in a flexible office-based workplace: Employee perceptions and priorities for change | Australia | Australia and New Zealand | Qualitative | Focus groups | Focus groups | Physical inactivity | Employees or managers | Employees; managers | Local |
| Palladino | 2018 | Changes in support for bans of illicit drugs, tobacco, and alcohol among adolescents and young adults in Europe, 2008-2014 | Multiple countries | Multiple countries | Quantitative | Cross-sectional study - representative | Repeat or serial survey - multiple waves | Preventive health | Children, adolescents or young adults | Adolescents; young adults | International |
| Parnell | 2019 | Attitudinal and behavioural responses to increasing tobacco control regulation among high smoking prevalence groups: A qualitative study | Australia | Australia and New Zealand | Qualitative | Interviews | Interviews | Tobacco use and smoking | Smokers or former smokers | Smokers or former smokers | Local |
| Patel | 2013 | Attitudes of business people to proposed smokefree shopping streets | New Zealand | Australia and New Zealand | Quantitative | Cross-sectional study - convenience or purposive | Single survey | Tobacco use and smoking | Policy actors, influencers or stakeholders | Retailers or industry | Local |
| Patterson | 2015 | A quantitative content analysis of UK newsprint coverage of proposed legislation to prohibit smoking in private vehicles carrying children | United Kingdom | UK and Ireland | Mixed methods | Media analysis | Media analysis | Tobacco use and smoking | News media | News media | National |
| Patterson | 2015 | Representations of minimum unit pricing for alcohol in UK newspapers: A case study of a public health policy debate | United Kingdom | UK and Ireland | Mixed methods | Media analysis | Media analysis | Alcohol use | News media | News media | National |
| Patwardhan | 2013 | Consumer perceptions of the sale of tobacco products in pharmacies and grocery stores among U.S. adults | United States | North America | Quantitative | Cross-sectional study - representative | Single survey | Tobacco use and smoking | General public | General public | National |
| Paul | 2013 | A cross-sectional survey of experts' opinions about the relative effectiveness of tobacco control strategies for the general population versus disadvantaged groups: What do we choose in the absence of evidence? | Multiple countries | Multiple countries | Quantitative | Cross-sectional study - convenience or purposive | Single survey | Tobacco use and smoking | Policy actors, influencers or stakeholders | Policy actors (eg. government officials) | International |
| Payán | 2017 | Advocacy coalitions involved in California's menu labeling policy debate: Exploring coalition structure, policy beliefs, resources, and strategies | United States | North America | Mixed methods | Multiple designs or methods | Document analysis; media analysis | Improve diet | Policy actors, influencers or stakeholders | Policy actors (eg. government officials) | State/regional |
| Pearson | 2013 | Public support for mandated nicotine reduction in cigarettes | United States | North America | Quantitative | Cross-sectional study - representative | Single survey | Tobacco use and smoking | General public | General public | National |
| Pell | 2019 | Support for, and perceived effectiveness of, the UK soft drinks industry levy among UK adults: Cross-sectional analysis of the International Food Policy Study | United Kingdom | UK and Ireland | Quantitative | Cross-sectional study - representative | Repeat or serial survey - single wave | Sugar-sweetened beverages | General public | General public | National |
| Pettigrew | 2012 | Public support for restrictions on fast food company sponsorship of community events | Australia | Australia and New Zealand | Quantitative | Cross-sectional study - representative | Single survey | Improve diet | General public | General public | State/regional |
| Pollard | 2013 | Public say food regulatory policies to improve health in Western Australia are important: Population survey results | Australia | Australia and New Zealand | Quantitative | Cross-sectional study - representative | Repeat or serial survey - multiple waves | Improve diet | General public | General public | State/regional |
| Porter | 2012 | Finding common ground: perspectives on community-based childhood obesity prevention | United States | North America | Mixed methods | Multiple designs or methods | Q methodology; survey; interviews | Overweight and obesity | General public | General public | Local |
| Puricelli Perin | 2014 | Perception of childhood obesity and support for prevention policies among latinos and whites | United States | North America | Quantitative | Cross-sectional study - convenience or purposive | Single survey | Overweight and obesity | General public | General public | Local |
| Purtle | 2018 | A Case Study of the Philadelphia Sugar-Sweetened Beverage Tax Policymaking Process: Implications for Policy Development and Advocacy | United States | North America | Qualitative | Interviews | Interviews | Sugar-sweetened beverages | Policy actors, influencers or stakeholders | Local councillors, city agency officials, advocate, news reporter, researchers | Local |
| Raine | 2014 | Understanding key influencers' attitudes and beliefs about healthy public policy change for obesity prevention | Canada | North America | Quantitative | Cross-sectional study - convenience or purposive | Single survey | Overweight and obesity | Policy actors, influencers or stakeholders | Policy actors (eg. government officials) | State/regional |
| Ravara | 2011 | Smoking behaviour predicts tobacco control attitudes in a high smoking prevalence hospital: a cross-sectional study in a Portuguese teaching hospital prior to the national smoking ban | Portugal | Europe | Quantitative | Cross-sectional study - convenience or purposive | Single survey | Tobacco use and smoking | Employees or managers | Employees | Local |
| Regan | 2016 | Perspectives of the public on reducing population salt intake in Ireland | Ireland | UK and Ireland | Quantitative | Cross-sectional study - representative | Single survey | Improve diet | General public | General public | National |
| Reindl | 2014 | Perceptions of College and University Presidents Regarding Tobacco-Free Campus Policies | United States | North America | Quantitative | Cross-sectional study - convenience or purposive | Single survey | Tobacco use and smoking | University/college students or staff | University/college students or staff | National |
| Reiter | 2012 | Appalachian residents' perspectives on new U.S. cigarette warning labels | United States | North America | Qualitative | Focus groups | Focus groups | Tobacco use and smoking | General public | General public | Local |
| Reiter | 2012 | Ohio Appalachian residents' views on smoke-free laws and cigarette warning labels | United States | North America | Qualitative | Focus groups | Focus groups | Tobacco use and smoking | General public | General public | Local |
| Richardson | 2019 | What young Australians think about a tax on sugar sweetened beverages | Australia | Australia and New Zealand | Quantitative | Cross-sectional study - convenience or purposive | Single survey | Sugar-sweetened beverages | Children, adolescents or young adults | Young adults | Local |
| Ries | 2011 | Newspaper reporting on legislative and policy interventions to address obesity: United States, Canada, and the United Kingdom | Multiple countries | Multiple countries | Qualitative | Media analysis | Media analysis | Overweight and obesity | News media | News media | International |
| Rissel | 2018 | Public support for bicycling and transport policies in Inner Sydney, Australia: A cross-sectional survey | Australia | Australia and New Zealand | Quantitative | Cohort study | Cross-sectional substudy | Physical inactivity | General public | General public | Local |
| Rivard | 2012 | Taxing sugar-sweetened beverages: a survey of knowledge, attitudes and behaviours | United States | North America | Quantitative | Cross-sectional study - representative | Single survey | Sugar-sweetened beverages | General public | General public | National |
| Robertson | 2015 | Regulating the sale of tobacco in New Zealand: A qualitative analysis of retailers' views and implications for advocacy | New Zealand | Australia and New Zealand | Qualitative | Interviews | Interviews | Tobacco use and smoking | Policy actors, influencers or stakeholders | Retailers or industry | National |
| Robertson | 2017 | New Zealand tobacco control experts' views towards policies to reduce tobacco availability | New Zealand | Australia and New Zealand | Qualitative | Interviews | Interviews | Tobacco use and smoking | Policy actors, influencers or stakeholders | Experts from academia; NGOs; health services & boards; former politicians. | National |
| Robles | 2017 | Predictors of public support for nutrition-focused policy, systems and environmental change strategies in Los Angeles County, 2013 | United States | North America | Quantitative | Cross-sectional study - representative | Single survey | Improve diet | General public | General public | Local |
| Roche | 2014 | Patrons' views about smoking in outdoor areas of licensed premises in South Australia: A pilot study | Australia | Australia and New Zealand | Quantitative | Cross-sectional study - convenience or purposive | Single survey | Tobacco use and smoking | General public | General public | Local |
| Rose | 2015 | Public Support for Family Smoking Prevention and Tobacco Control Act Point-of-Sale Provisions: Results of a National Study | United States | North America | Quantitative | Cross-sectional study - representative | Single survey | Tobacco use and smoking | General public | General public | National |
| Rosenberg | 2012 | Public support for tobacco control policy extensions in Western Australia: A cross-sectional study | Australia | Australia and New Zealand | Quantitative | Cross-sectional study - representative | Single survey | Tobacco use and smoking | General public | General public | State/regional |
| Ruokolainen | 2018 | Social climate on tobacco control in an advanced tobacco control country: A population-based study in Finland | Finland | Europe | Quantitative | Cohort study | Cross-sectional substudy | Tobacco use and smoking | General public | General public | National |
| Russell | 2020 | The political construction of public health nutrition problems: a framing analysis of parliamentary debates on junk-food marketing to children in Australia | Australia | Australia and New Zealand | Qualitative | Document or submission analysis | Document analysis | Improve diet | Policy actors, influencers or stakeholders | Political representatives | National |
| Sæbø | 2019 | Children's right to smoke-free air: Public support in Norway for banning smoking in vehicles with children present | Norway | Europe | Quantitative | Cross-sectional study - representative | Single survey | Tobacco use and smoking | General public | General public | National |
| Sainsbury | 2018 | Public support for government regulatory interventions for overweight and obesity in Australia | Australia | Australia and New Zealand | Quantitative | Cross-sectional study - representative | Single survey | Overweight and obesity | General public | General public | National |
| Sansone | 2020 | Secondhand smoke exposure in public places and support for smoke-free laws in Japan: Findings from the 2018 ITC Japan survey | Japan | Asia | Quantitative | Cohort study | Cross-sectional substudy | Tobacco use and smoking | Multiple groups | General public; smokers or former smokers | National |
| Schmidt | 2014 | Communicating program outcomes to encourage policymaker support for evidence-based state tobacco control | United States | North America | Qualitative | Interviews | Interviews | Tobacco use and smoking | Policy actors, influencers or stakeholders | Former politicians; health NGO advocates/lobbyists. | State/regional |
| Schmidt | 2016 | Secondhand Smoke Exposure and Smoke-Free Policy Support Among Public Housing Authority Residents in Rural and Tribal Settings | United States | North America | Quantitative | Cross-sectional study - convenience or purposive | Single survey | Tobacco use and smoking | Other community group | Public housing residents | State/regional |
| Schmidt | 2018 | Attitudes towards potential new tobacco control regulations among U.S. adults | United States | North America | Quantitative | Cross-sectional study - representative | Single survey | Tobacco use and smoking | General public | General public | National |
| Schmitt | 2014 | Public and policy maker support for point-of-sale tobacco policies in New York | United States | North America | Quantitative | Cross-sectional study - representative | Single survey | Tobacco use and smoking | Multiple groups | General public; policymakers | State/regional |
| Schmitt | 2015 | Support for a ban on tobacco powerwalls and other point-of-sale displays: Findings from focus groups | United States | North America | Qualitative | Focus groups | Focus groups | Tobacco use and smoking | General public | General public | Local |
| Schmitt | 2017 | Public and Opinion Leader Willingness to Fund Obesity-Focused Policies in Kansas | United States | North America | Quantitative | Cross-sectional study - convenience or purposive | Single survey | Overweight and obesity | Multiple groups | General public; opinion leaders | State/regional |
| Schmitt | 2018 | Relationships between theoretically derived short-term outcomes and support for policy among the public and decision- makers | United States | North America | Quantitative | Cross-sectional study - convenience or purposive | Single survey | Overweight and obesity | Multiple groups | General public; opinion leaders | State/regional |
| Schreuders | 2018 | To what extent and why adolescents do or do not support future tobacco control measures: A multimethod study in the Netherlands | The Netherlands | Europe | Mixed methods | Multiple designs or methods | Cross-sectional survey; focus groups | Tobacco use and smoking | Children, adolescents or young adults | Children or adolescents | Local |
| Schulte | 2016 | Belief in Food Addiction and Obesity-Related Policy Support | United States | North America | Quantitative | Cross-sectional study - convenience or purposive | Single survey | Overweight and obesity | General public | General public | National |
| Seo | 2011 | The effect of a smoke-free campus policy on college students' smoking behaviors and attitudes | United States | North America | Quantitative | Cohort study | Cohort study | Tobacco use and smoking | University/college students or staff | University/college students or staff | Local |
| Seo | 2015 | Korean public opinion on alcohol control policy: A cross-sectional International Alcohol Control study | South Korea | Asia | Quantitative | Cross-sectional study - representative | Repeat or serial survey - single wave | Alcohol use | General public | General public | National |
| Sharman | 2019 | Acceptability and perceived feasibility of strategies to increase public transport use for physical activity gain - A mixed methods study | Australia | Australia and New Zealand | Mixed methods | Multiple designs or methods | Cross-sectional survey; focus groups; interviews | Physical inactivity | General public | General public | State/regional |
| Signal | 2012 | Front-of-pack nutrition labelling in New Zealand: an exploration of stakeholder views about research and implementation | New Zealand | Australia and New Zealand | Qualitative | Interviews | Interviews | Improve diet | Policy actors, influencers or stakeholders | Stakeholders - food industry representatives; policymakers; NGO representatives. | National |
| Signal | 2018 | Appetite for health-related food taxes: New Zealand stakeholder views | New Zealand | Australia and New Zealand | Qualitative | Interviews | Interviews | Improve diet | Policy actors, influencers or stakeholders | Politicians; government officials; food industry; public health experts; consumer groups | National |
| Signal | 2019 | Prime minister for a day: Children’s views on junk food marketing and what to do about it | New Zealand | Australia and New Zealand | Qualitative | Interviews | Interviews | Improve diet | Children, adolescents or young adults | Children or adolescents | Local |
| Sikorski | 2012 | Public attitudes towards prevention of obesity | Germany | Europe | Quantitative | Cross-sectional study - representative | Single survey | Overweight and obesity | General public | General public | National |
| Simon | 2014 | Public opinion on nutrition-related policies to combat child obesity, Los Angeles County, 2011 | United States | North America | Quantitative | Cross-sectional study - representative | Repeat or serial survey - single wave | Overweight and obesity | General public | General public | Local |
| Smith | 2016 | Tobacco Pricing in Military Stores: Views of Military Policy Leaders | United States | North America | Qualitative | Interviews | Interviews | Tobacco use and smoking | Policy actors, influencers or stakeholders | Policy actors (eg. government officials) | National |
| Sohlberg | 2019 | In favour of tobacco control? Former smokers’ support for tobacco policies | Sweden | Europe | Quantitative | Cohort study | Cross-sectional substudy | Tobacco use and smoking | Smokers or former smokers | Smokers or former smokers | National |
| Somerville | 2015 | Public attitudes towards pricing policies to change health-related behaviours: A UK focus group study | United Kingdom | UK and Ireland | Qualitative | Focus groups | Focus groups | Preventive health | General public | General public | Local |
| Sonnenberg | 2020 | Support for Aggressive Tobacco Control Interventions Among California Adolescents and Young Adults | United States | North America | Quantitative | Cross-sectional study - representative | Repeat or serial survey - single wave | Tobacco use and smoking | Children, adolescents or young adults | Children or adolescents | State/regional |
| Spivak | 2018 | Self-interest and public opinion in health policy: Smoking behavior and support for tobacco control | United States | North America | Quantitative | Cross-sectional study - representative | Single survey | Tobacco use and smoking | General public | General public | State/regional |
| Stanesby | 2017 | Experience of harm from others’ drinking and support for stricter alcohol policies: Analysis of the Australian National Drug Strategy Household Survey | Australia | Australia and New Zealand | Quantitative | Cross-sectional study - representative | Repeat or serial survey - single wave | Alcohol use | General public | General public | National |
| Stephens | 2015 | Nutrition promotion approaches preferred by Australian adolescents attending schools in disadvantaged neighbourhoods: A qualitative study | Australia | Australia and New Zealand | Qualitative | Interviews | Interviews | Improve diet | Children, adolescents or young adults | Children or adolescents | Local |
| Stok | 2016 | Hungry for an intervention? Adolescent's ratings of acceptability of eating-related intervention strategies | Multiple countries | Multiple countries | Quantitative | Cross-sectional study - convenience or purposive | Single survey | Improve diet | Children, adolescents or young adults | Children or adolescents | International |
| Storvoll | 2014 | Changes in attitudes towards restrictive alcohol policy measures: The mediating role of changes in beliefs | Norway | Europe | Quantitative | Cross-sectional study - representative | Repeat or serial survey - multiple waves | Alcohol use | General public | General public | National |
| Storvoll | 2015 | Predicting attitudes toward a restrictive alcohol policy: Using a model of distal and proximal predictors | Norway | Europe | Quantitative | Cross-sectional study - representative | Single survey | Alcohol use | General public | General public | National |
| Street | 2017 | Community perspectives on the use of regulation and law for obesity prevention in children: A citizens’ jury | Australia | Australia and New Zealand | Qualitative | Deliberative | Citizens' jury | Overweight and obesity | General public | General public | State/regional |
| Street | 2018 | Supporting youth wellbeing with a focus on eating well and being active: views from an Aboriginal community deliberative forum | Australia | Australia and New Zealand | Qualitative | Deliberative | Citizens' jury | Preventive health | Other community group | Aboriginal population | Local |
| Sugerman | 2011 | Using an opinion poll to build an obesity-prevention social marketing campaign for low-income Asian and Hispanic immigrants: report of findings | United States | North America | Quantitative | Cross-sectional study - representative | Single survey | Overweight and obesity | Other community group | CALD and migrant low income population | Local |
| Suggs | 2011 | European Union public opinion on policy measures to address childhood overweight and obesity | Multiple countries | Multiple countries | Quantitative | Cross-sectional study - representative | Repeat or serial survey - single wave | Overweight and obesity | General public | General public | International |
| Sureda | 2015 | Secondhand smoke in outdoor settings: Smokers' consumption, non-smokers' perceptions, and attitudes towards smoke-free legislation in Spain | Spain | Europe | Quantitative | Cross-sectional study - representative | Single survey | Tobacco use and smoking | General public | General public | Local |
| Swift | 2015 | Australian smokers' support for plain or standardised packs before and after implementation: findings from the ITC Four Country Survey | Australia | Australia and New Zealand | Quantitative | Cohort study | Cohort study | Tobacco use and smoking | Smokers or former smokers | Smokers or former smokers | National |
| Tabak | 2013 | Policy perceptions related to physical activity and healthy eating in Mississippi | United States | North America | Quantitative | Cross-sectional study - representative | Single survey | Preventive health | General public | General public | Local |
| Tapp | 2016 | Great Britain adults' opinions on cycling: Implications for policy | United Kingdom | UK and Ireland | Quantitative | Cross-sectional study - representative | Repeat or serial survey - multiple waves | Physical inactivity | General public | General public | National |
| Thomas-Meyer | 2017 | Public responses to proposals for a tax on sugar-sweetened beverages: A thematic analysis of online reader comments posted on major UK news websites | United Kingdom | UK and Ireland | Qualitative | Media analysis | Reader comment analysis | Sugar-sweetened beverages | News media | Reader commentary on online news articles | National |
| Thrasher | 2014 | Print media coverage around failed and successful tobacco tax initiatives: the South Carolina experience | United States | North America | Mixed methods | Media analysis | Media analysis | Tobacco use and smoking | News media | News media | State/regional |
| Tindall | 2016 | Alcohol-related crime in city entertainment precincts: Public perception and experience of alcohol-related crime and support for strategies to reduce such crime | Australia | Australia and New Zealand | Quantitative | Cross-sectional study - convenience or purposive | Single survey | Alcohol use | General public | General public | State/regional |
| Tobin | 2012 | Support for breaking the nexus between alcohol and community sports settings: Findings from the VicHealth Community Attitudes Survey in Australia | Australia | Australia and New Zealand | Quantitative | Cross-sectional study - representative | Single survey | Alcohol use | General public | General public | State/regional |
| Trainer | 2017 | Public perceptions of the tobacco-free generation in Tasmania: adults and adolescents | Australia | Australia and New Zealand | Quantitative | Cross-sectional study - representative | Single survey | Tobacco use and smoking | Multiple groups | General public; adolescents | State/regional |
| Tynan | 2019 | Attitudes Toward Smoke-Free Casino Policies Among US Adults, 2017 | United States | North America | Quantitative | Cross-sectional study - representative | Repeat or serial survey - single wave | Tobacco use and smoking | General public | General public | National |
| van der Sar | 2011 | The opinion of adolescents and adults on Dutch restrictive and educational alcohol policy measures | The Netherlands | Europe | Quantitative | Cross-sectional study - representative | Single survey | Alcohol use | General public | General public | National |
| van der Sar | 2012 | Dutch and Norwegian support of alcohol policy measures to prevent young people from problematic drinking: A cross-national comparison | Multiple countries | Multiple countries | Quantitative | Cross-sectional study - representative | Single survey | Alcohol use | General public | General public | International |
| Van Hal | 2018 | European University Students' Experiences and Attitudes toward Campus Alcohol Policy: A Qualitative Study | Multiple countries | Multiple countries | Qualitative | Focus groups | Focus groups | Alcohol use | University/college students or staff | University/college students or staff | International |
| van Mourik | 2018 | Support for a point-of-sale cigarette display ban among smokers: findings from the international tobacco control (ITC) Netherlands survey | The Netherlands | Europe | Quantitative | Cohort study | Cohort study | Tobacco use and smoking | Smokers or former smokers | Smokers or former smokers | National |
| Watson | 2017 | Support for food policy initiatives is associated with knowledge of obesity-related cancer risk factors | Australia | Australia and New Zealand | Quantitative | Cross-sectional study - convenience or purposive | Single survey | Improve diet | General public | General public | State/regional |
| Welch | 2012 | State legislators' support for evidence-based obesity reduction policies | United States | North America | Quantitative | Cross-sectional study - convenience or purposive | Single survey | Overweight and obesity | Policy actors, influencers or stakeholders | Political representatives | National |
| Whyte | 2014 | Advancing the retail endgame: Public perceptions of retail policy interventions | New Zealand | Australia and New Zealand | Quantitative | Cross-sectional study - representative | Single survey | Tobacco use and smoking | General public | General public | National |
| Winickoff | 2016 | Public support for raising the age of sale for tobacco to 21 in the United States | United States | North America | Quantitative | Cross-sectional study - representative | Repeat or serial survey - single wave | Tobacco use and smoking | General public | General public | National |
| Wolfson | 2017 | Perspectives on learning to cook and public support for cooking education policies in the United States: A mixed methods study | United States | North America | Mixed methods | Multiple designs or methods | Cross-sectional survey; focus groups | Improve diet | General public | General public | Local |
| Worsley | 2011 | Australian consumers' views of fruit and vegetable policy options | Australia | Australia and New Zealand | Quantitative | Cross-sectional study - representative | Single survey | Improve diet | General public | General public | State/regional |
| Worsley | 2015 | Food concerns and support for environmental food policies and purchasing | Australia | Australia and New Zealand | Quantitative | Cross-sectional study - representative | Single survey | Improve diet | General public | General public | National |
| Yun | 2016 | Public opinions on disclosure of tobacco components: Results of a nationwide cross-sectional survey in Republic of Korea | South Korea | Asia | Quantitative | Cross-sectional study - representative | Single survey | Tobacco use and smoking | General public | General public | National |
| Yun | 2018 | Assessing the social climate of physical (in)activity in Canada | Canada | North America | Quantitative | Cross-sectional study - representative | Single survey | Physical inactivity | General public | General public | National |
| Yun | 2019 | Political Orientation and Public Attributions for the Causes and Solutions of Physical Inactivity in Canada: Implications for Policy Support | Canada | North America | Quantitative | Cross-sectional study - representative | Single survey | Physical inactivity | General public | General public | National |
| Zatonski | 2018 | Characterising smokers of menthol and flavoured cigarettes, their attitudes towards tobacco regulation, and the anticipated impact of the Tobacco Products Directive on their smoking and quitting behaviours: The EUREST-PLUS ITC Europe Surveys | Multiple countries | Multiple countries | Quantitative | Cohort study | Cross-sectional substudy | Tobacco use and smoking | Smokers or former smokers | Smokers or former smokers | International |
| Zollinger | 2012 | Effect of personal characteristics on individual support for indoor smoke-free air laws, Indiana, 2008 | United States | North America | Quantitative | Cross-sectional study - representative | Single survey | Tobacco use and smoking | General public | General public | State/regional |
